# Impact of macronutrient supplements for children born preterm or small for gestational age on developmental and metabolic outcomes: A systematic review and meta-analysis

**DOI:** 10.1371/journal.pmed.1002952

**Published:** 2019-10-30

**Authors:** Luling Lin, Emma Amissah, Gregory D. Gamble, Caroline A. Crowther, Jane E. Harding

**Affiliations:** Liggins Institute, University of Auckland, Auckland, New Zealand; London School of Hygiene and Tropical Medicine, UNITED KINGDOM

## Abstract

**Background:**

Nutritional supplements may improve development of infants born small (preterm or small for gestational age [SGA]) but may increase the risk of later metabolic disease. We conducted a systematic review and meta-analysis to assess the effects of macronutrient supplements for infants born small on later development and metabolism.

**Methods and findings:**

We searched OvidMedline, Embase, Cochrane CENTRAL, and Cochrane Database of Systematic Reviews from inception to April 1, 2019, and controlled-trials.com, clinicaltrials.gov, and anzctr.org.au. Randomised or quasirandomised trials were included if the intention was to increase macronutrient intake to improve growth or development of infants born small and assessed post-discharge outcomes. Co-primary outcomes were cognitive impairment and metabolic risk, evaluated in toddlers (<3 years), childhood (3 to 8 years), and adolescence (9 to 18 years). Two reviewers independently extracted data. Quality was assessed using the Cochrane Risk of Bias tool, and data were pooled using random-effect models.

Twenty-one randomised and one quasirandomised trial of variable methodological quality involving 3,680 infants were included. In toddlers born small, supplementation did not alter cognitive impairment (relative risk [RR] 1.00; 95% confidence interval [CI] 0.67 to 1.49; *P* = 0.99), and there were no differences in cognitive scores (mean difference [MD] 0.57; 95% CI −0.71 to 1.84; *P* = 0.38) or motor scores (MD 1.16; 95% CI −0.32 to 2.65; *P* = 0.12) between supplemented and unsupplemented groups. However, fewer supplemented children had motor impairment (RR 0.76; 95% CI 0.62 to 0.94; *P* = 0.01). In subgroup analyses, supplementation improved cognitive scores in boys (MD 5.60; 95% CI 1.07 to 10.14; *P* = 0.02), but not girls born small (MD −2.04; 95% CI −7.04 to 2.95; *P* = 0.42), and did not alter cognitive or motor scores in the subgroup of children born SGA. In childhood, there was no difference in cognitive impairment (RR 0.81; 95% CI 0.26 to 2.57; *P* = 0.72) or cognitive scores (MD 1.02; 95% CI −1.91 to 3.95; *P* = 0.50) between supplemented and unsupplemented groups. There were also no differences in blood pressure, triglyceride, and low-density lipoprotein (LDL) concentrations (all *P* > 0.05). However, supplemented children had lower fasting glucose (mmol/L: MD −0.20; 95% CI −0.34 to −0.06; *P* = 0.005) and higher high-density lipoprotein (HDL) concentrations (mmol/L: MD 0.11; 95% CI 0.02 to 0.19; *P* = 0.02). In subgroup analyses, there was no evidence of differences in blood pressure between supplemented and unsupplemented groups in boys or girls born small, or in SGA children. In adolescence, there was no difference between supplemented and unsupplemented groups in blood pressure, triglycerides, LDL and HDL concentrations, fasting blood glucose, insulin resistance, and fasting insulin concentrations (all *P* > 0.05). Limitations include considerable unexplained heterogeneity, low to very low quality of the evidence, and limited data beyond early childhood.

**Conclusions:**

In this systematic review and meta-analysis of randomised trials, we found no evidence that early macronutrient supplementation for infants born small altered later cognitive function, although there was some evidence that supplementation may decrease motor impairment in toddlers. Contrary to the findings from observational studies, evidence from randomised trials suggests that early macronutrient supplementation for infants born small improves some metabolic outcomes in childhood.

**PROSPERO registration:**

CRD42019127858.

## Abstract 2

Jane Harding and colleagues reveal no cognitive benefit later in life to premature babies that receive macronutirient supplements following birth.

## Author summary

**Why was this study done?:**

- Preterm and small-for-gestational-age (SGA) infants are at increased risk of poor growth, delayed development, and disability.
- Nutritional supplements are often given to these infants to improve growth and development, but observational studies suggest that this may lead to later increased risk of metabolic disease.
- The balance of these potential later benefits and risks is not known, and it is possible that they may be different in girls and boys.

**What did the researchers do and find?:**

- We undertook a systematic review and meta-analysis of 21 randomised clinical trials and 1 quasirandomised trial of nutritional supplements involving 3,680 infants born preterm or SGA.
- We found that early supplements given to infants born small did not alter overall cognitive function, but in toddlers, early supplements decreased motor impairment and improved cognitive scores in boys.
- Some markers of risk of metabolic disease were improved after early nutritional supplements.

**What do these findings mean?:**

- Early nutritional supplements for infants born preterm or small have little overall benefit for cognitive development but also do not appear to increase metabolic risk.
- These findings from randomised trials are in contrast to those from previous observational studies.
- Despite large numbers of trials involving thousands of infants, there is still limited evidence about the benefits and risks of early nutritional supplements after early childhood.

## Introduction

Infants born preterm or small for gestational age (SGA) are at increased risk of poor growth, developmental delay, and disability [1–4]. As adults, they are at increased risk of obesity, diabetes, and cardiovascular disease [5]. Providing preterm and SGA infants with higher protein and energy intake during the first few weeks after birth has been associated with improved short-term growth and better developmental outcomes from infancy to adolescence [6–12]. However, observational data suggest that there may be important tradeoffs between early cognitive development and later metabolic diseases in preterm infants [13]. Rapid body mass index (BMI) gain and linear growth are associated with better cognitive development but at the expense of increased risk for adiposity and metabolic and cardiovascular disease in adulthood [14,15]. There is also limited evidence that these effects may differ in girls and boys [16].

Three previous systematic reviews have compared the effect of supplemented versus unsupplemented formula started after hospital discharge, fortified versus unfortified breastmilk started in hospital or after hospital discharge [11,17,18]. None of these reviews reported developmental outcomes after 18 months of age, and none reported long-term metabolic outcomes or assessed potential sex-specific effects.

We therefore undertook a systematic review and meta-analysis to assess the published data from randomised trials on the effects of early macronutrient supplements fed to preterm and SGA infants on developmental and metabolic outcomes after hospital discharge, and also whether these effects differed in girls and boys.

## Methods

We used Preferred Reporting Items for Systematic Reviews and Meta-Analyses (PRISMA) guidelines and registered this review prospectively in PROSPERO (registration number CRD42019127858).

### Search strategy and selection criteria

We searched OvidMedline, Embase, Cochrane Library Central Registry of Controlled Trials, and Cochrane Database of Systematic Reviews from inception to April 1, 2019. We searched for eligible ongoing trials in Current Controlled Trials (www.controlled-trials.com), Clinical Trials (www.clinicaltrials.gov), and the Australian and New Zealand Clinical Trials Registry (www.anzctr.org.au). Conference abstracts were included if they provided usable data.

Inclusion criteria were as follows: (1) randomised controlled trials (RCTs) and quasi-RCTs without restrictions on date of publication or language; (2) infants born preterm (<37 weeks’ gestation) or small (birth weight <2.5 kg or <10th centile); (3) the intervention was intended to increase intake of one or more macronutrients (protein, carbohydrate, fat, energy, or protein to energy ratio) with the primary aim of improving growth or development (interventions could be enteral, parenteral, or both; commence any time during initial hospitalisation after birth or after discharge; and must have been provided for ≥1 week); and (4) reported any of the prespecified outcomes (S1 Appendix) assessed after term equivalent age (>37 weeks’ gestation) or after discharge from hospital after birth.

Studies that reported comparisons between unsupplemented and supplemented nutrition with parenteral supplements, human breast milk supplements, formula milk, or other macronutrients were eligible for inclusion. We excluded trials comparing the timing of the introduction of nutrition (early versus delayed feeding); macronutrients of different composition (e.g., different types of lipids or proteins); variations in intakes of micronutrients (including sodium, potassium, calcium, phosphorous, vitamins, other minerals, amino acids, fatty acids); or focussed on gastrointestinal development.

Co-primary outcomes were cognitive impairment (below −1 standard deviation [SD] on standard tests of cognitive development [toddlers] or cognition/intelligence quotient [later ages] or as defined by trialist) and any metabolic risk (any of the following defined by trialists: overweight/obese, increased waist circumference, increased fat mass or fat mass percentage, elevated plasma triglyceride concentrations, low high-density lipoprotein [HDL] concentrations, elevated low-density lipoprotein [LDL] concentrations, elevated fasting plasma glucose concentrations, insulin resistance, impaired glucose tolerance, diagnosis of type 2 diabetes, high blood pressure, impaired flow-mediated vasodilatation) (full list of outcomes in S1 Appendix). The outcomes were evaluated in toddlers (<3 years), childhood (3 to 8 years), and adolescence (9 to 18 years).

### Data collection and analysis

Two reviewers (LL and EA) independently screened titles and abstracts of all records identified, assessed potentially eligible full-text articles for inclusion, extracted data into a template data extraction form, and assessed the risk of bias for included studies using Cochrane criteria [19]. Discrepancies were resolved by discussion or with a third author (JH).

We assessed risk of bias for each key outcome using the Grading of Recommendations Assessment, Development and Evaluation (GRADE) [20] approach and created a “Summary of findings” table using the GradePro Guideline Development Tool (GDT; https://gradepro.org/). If a trial reported the same outcomes measured at different time points in childhood or beyond (>3 years), we chose the age group with the most data for assessment of the quality of evidence. We assessed quality of evidence for developmental outcomes for the following: composite of survival free of disability, cerebral palsy in toddlers, cognitive impairment in toddlers, cognitive scores in toddlers, motor impairment in toddlers, motor development scores in toddlers, and school performance. We assessed quality of evidence for metabolic outcomes for the following: overweight/obesity, triglyceride concentrations, HDL concentrations, LDL concentrations, systolic blood pressure (SBP), elevated fasting plasma glucose concentrations, and insulin resistance (all at >3 years).

### Statistical analysis

We undertook meta-analyses using RevMan 5.3 [21] using random-effects models and calculated relative risks (RRs) and mean differences (MDs) with 95% confidence intervals [CIs]. *P* < 0.05 denoted statistical significance for all models, and this critical value was not split for each of the co-primary outcomes. We calculated *I*^2^ and χ^2^ tests to determine statistical heterogeneity, with *I*^2^ > 50% and *P* < 0.10 considered significant heterogeneity. We assessed potential bias due to small study effects by visual inspection of funnel plots when there were more than 10 trials. We planned to conduct sensitivity analyses for GRADE outcomes by examining only trials considered to have low risk of selection and detection bias. We conducted subgroup analyses to explore whether the effects of supplements differed with sex, SGA, or timing of supplementation and tested for interactions for GRADE outcomes. No unplanned analyses were performed.

## Results

**Fig 1:**
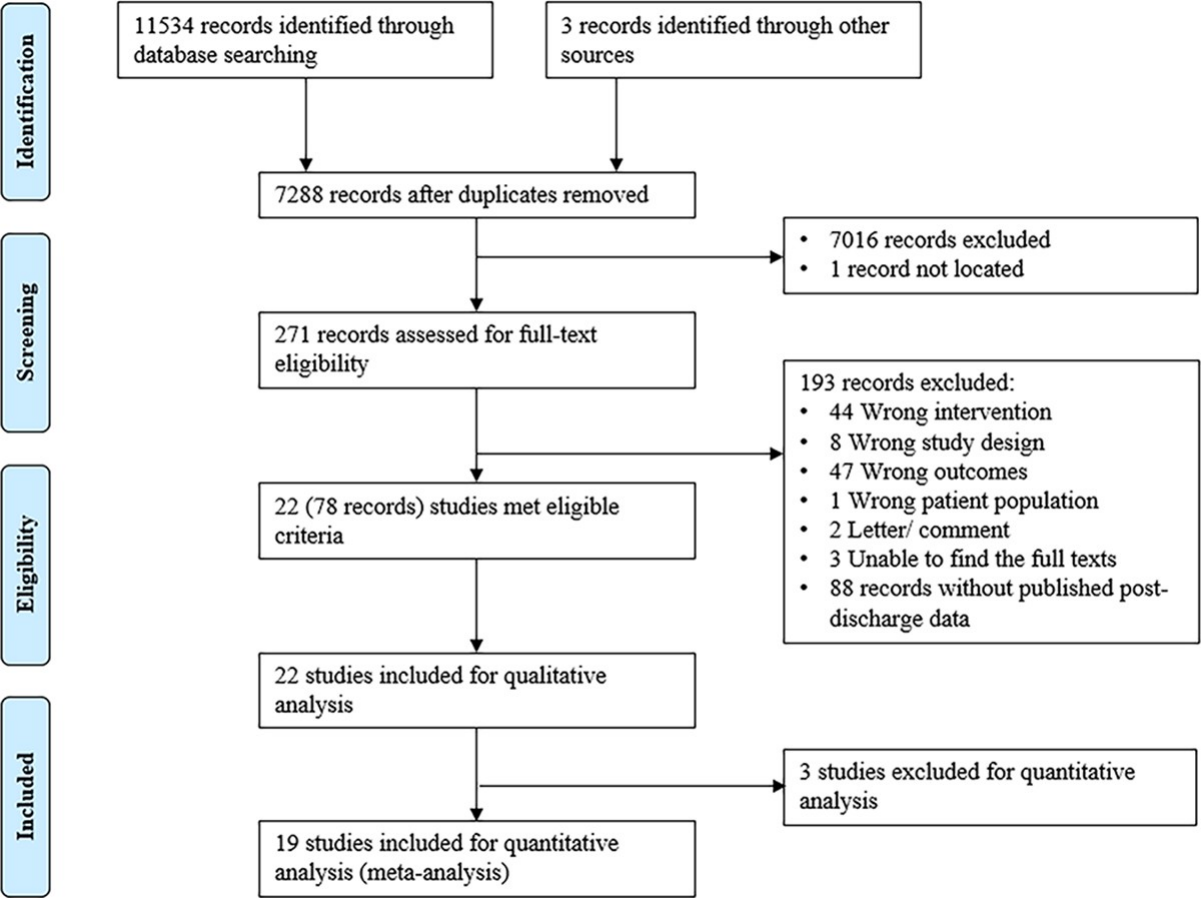
Flow diagram for included studies.

After de-duplication, 7,288 records were identified. After title and abstract screening, we completed full-text screening for 271 records. We excluded 193 records that did not meet our inclusion criteria. We included the remaining 21 RCTs and 1 quasi-RCT with 3,680 infants in the qualitative analysis and 19 RCTs with 3,172 infants in the quantitative analysis (Fig 1). The included infants were born between 1963 and 2017. One study included term SGA infants [22], and the remaining 21 studies included preterm infants. Supplements were given in hospital in 10 studies [6,16,23–30], post discharge in 10 studies [31–40], and both in hospital and post discharge in 2 studies [22,41] (Table 1).

**Table 1:** Characteristics of included studies.

Author/Year	Country	Participants	Participants, *n*	Intervention	Control	Duration	Outcomes
Agosti 2003 [31]	Italy	Inclusion criteria: preterm BW < 1,500 g and previously fed with a preterm formula Exclusion criteria: malformations intraventricular hemorrhage, periventricular leukomalacia, chronic lung disease, necrotizing enterocolitis grade >1, total parenteral nutrition >2 weeks, sepsis, retinopathy of prematurity grade >1	Intervention: 69 Control: 52	Preterm formula (protein 2.4 g/100 ml, energy 80 kcal/100 ml)	Standard term formula (protein 1.7 g/100 ml, energy 70 kcal/100 ml)	Started from 40 weeks PMA, stopped at 55 weeks PMA	GMDS at 6, 9, and 12 months’ CA (Data presented in figures, no SD reported).
Amesz 2010 [32]	The Netherlands	Inclusion criteria: preterm GA ≤ 32 weeks or BW ≤ 1,500 g Exclusion criteria: congenital malformations or conditions known to affect growth or body composition (e.g., severe bronchopulmonary dysplasia, an inborn error of metabolism, cardiac or renal disease, necrotizing enterocolitis with substantial gut loss, grade 4 intraventricular hemorrhage)	Intervention: 52 Control: 50	Postdischarge formula (protein 1.7 g/100 ml, fat 3.5 g/100 ml, carbohydrate 7.0 g/100 ml, energy 67 kcal/100 ml)	Term formula (protein 1.47 g/100 ml, fat 3.5 g/100 ml, carbohydrate 7.2 g/100 ml, energy 70 kcal/100 ml)	Started from term, stopped at 6 months’ CA	Blood pressure, triglycerides, HDL, LDL, fasting blood glucose concentration, insulin sensitivity, insulin resistance (HOMA-IR), fasting leptin at 8 years’ CA.
Bellagamba 2016 [23]	Italy	Inclusion criteria: preterm BW between 500 and 1,249 g	Intervention: 82 Control: 82	High protein intake group (protein supplementation started at 1.5 g/kg/day and increased by 0.5 g/kg/day to a maximum of 3.5 g/kg/day on the fifth day after birth)	Standard protein intake group (protein supplementation started at 1.5g/kg/day and increased by 0.5 g/kg/day to a maximum of 2.5 g/kg/day on the third day after birth)	Started from birth, stopped at discharge	Bayley III at 2 years’ CA.
Biasini 2012 [24]	Italy	Inclusion criteria: preterm BW between 580 and 1,250 g and GA < 32 weeks	Intervention: 34 Control: 27	Protein supplemented group (protein 4.8 g/kg/day, energy 141 kcal/day)	Control group (protein 3.5 g/kg/day, energy 135 kcal/day)	Started from the first day of full enteral feeding, stopped at discharge	GMDS at 12 and 18 months’ CA; GMDS at 18 and 24 months’ CA for SGA infants.
Cooke 2001 [33]	UK	Inclusion criteria: preterm GA ≤ 34 weeks and BW ≤ 1,750 g, and growing normally at the time of hospital discharge, i.e., ≥25 g/d Exclusion criteria: systemic disease or require medication	Intervention: 56 Control: 57	Preterm formula (protein 2.2 g/100 ml, fat 4.4 g/100 ml, carbohydrate 8.5 g/100 ml, energy 80 kcal/100 ml)	Term formula (protein 1.4 g/100 ml, fat 3.6 g/100 ml, carbohydrate 7.5 g/100 ml, energy 66 kcal/100 ml)	Started from discharge, stopped at 6 months’ CA	Bayley II at 18 months’ CA.
Cooper 1988 [25]	South Africa	Inclusion criteria: preterm GA < 36 weeks and BW < 1,600 g Exclusion criteria: major congenital abnormalities, congenital infections or severe intrauterine growth retardation, or withdrawn from the study (if intake of own mother’s milk was > 100 kcal/kg/d)	Intervention: 10 Control: 10	Preterm formula (protein 2.2 g/100 ml, fat 3.66 g/100 ml, carbohydrate 8.6 g/100 ml, energy 75 kcal/100 ml)	Standard formula (protein 1.6 g/100 ml, fat 3.4 g/100 ml, carbohydrate 7.4 g/100 ml, energy 67 kcal/100 ml)	Started from the day that half the caloric intake was enteral and stopped after 5 weeks or when infants reached 2,000 g	GMDS at 12 months’ and 3 years’ CA.
da Cunha 2016 [34]	Brazil	Inclusion criteria: preterm GA < 37 weeks and BW < 1,500 g, and discharged exclusively breastfeeding Exclusion criteria: major malformations; hydrocephalus; chromosomal abnormalities; fetal hydrops; congenital infections; maternal use of illicit drugs, tobacco, alcohol, and continuous use of corticosteroids; twin pregnancy; necrotizing enterocolitis sequelae; cerebral palsy	Intervention: 27 Control: 27	Breast milk supplementation (daily increase of 0.56 g of protein, 1.04 g of total fat, and 2.12 g of carbohydrates)	Breast milk without supplementation	Started 7–10 days after discharge, stopped at 4 to 6 months	Bayley III at 12 months’ CA
Dogra 2017 [26]	India	Inclusion criteria: preterm GA < 32 weeks Exclusion criteria: lethal congenital malformations	Intervention: 59 Control: 56	Fortified breast milk with higher enteral protein intake (fortifier containing protein 1.0 g/100 mL, fat 0.01 g/100 mL, carbohydrate 3.6 g/100 mL, energy 17.2 kcal/100 mL)	Fortified breast milk with standard enteral protein intake (fortifier containing protein 0.4 g/100 mL, fat 0.2 g/100 mL, carbohydrate 2.4 g/100 mL, energy 13 kcal/100 mL)	Started when infants reached a feed volume of 100 mL/kg/ day, stopped at discharge or full breastfeeds, whichever was earlier	DASII at 12 to 18 months’ CA
Fewtrell 2001 [22]	UK	Inclusion criteria: GA ≥ 37 weeks and BW below the 10th centile for gestation and sex according to UK growth charts	Intervention: 152 Control: 147	Enriched formula (protein 1.85 g/100 mL, fat 3.96 g/100 mL, carbohydrate 7.24 g/100 mL, energy 72 kcal/100 mL)	Term formula (protein 1.45 g/100 mL, fat 3.85 g/100 mL, carbohydrate 6.96 g/100 mL, energy 68 kcal/100 mL)	Started within the first week, stopped at 9 months’ CA	Bayley II at 9 and 18 months’ CA; blood pressure at 6 to 8 years’ CA
Friel 1993 [35]	Canada	Inclusion criteria: BW < 1,500 g Exclusion criteria: breast-fed, hydrocephalus, liver dysfunction, or any congenital malformations	Intervention: 27 Control: 27	Low BW formula (protein 1.73 g/100 mL, fat 3.7 g/100 mL, carbohydrate 7.1 g/100 mL, energy 67 kcal/100 mL)	Term formula (protein 1.57 g/100 mL, fat 3.6 g/100 mL, carbohydrate 7.3 g/100 mL, energy 67 kcal/100 mL)	Started when the infants reached a weight of 1,850 ± 100 g, stopped at 5 months after discharge	GMDS at 3, 6, 9, 12 months’ CA (Data presented in figures, no SD reported).
Goldman 1969 [27]	US	Inclusion criteria: BW < 2,000 g Exclusion criteria: major congenital malformations, intestinal obstruction, Rhesus disease, >3 days old on admission, or died during the first few days generally received no milk feedings	Intervention: 152 Control: 152	Enriched formula (protein 4.0 g/100 mL, fat 3.9 g/100 mL, carbohydrate 7.6 g/10 mml, 80 kcal/100 mL)	Standard formula (protein 2.0 g/100 mL, fat 3.9 g/100 mL, carbohydrate 9.6 g/100 mL, energy 80 kcal/100 mL)	Started from 24 to 72 hours, stopped when the infants reached 2,200 g (at discharge)	Cognitive impairment (Stanford-Binet scores) at 3 years’ CA.
Jeon 2011 [36]	Korea	Inclusion criteria: preterm GA < 33 weeks and BW < 1,500 g, formula as the primary food source Exclusion criteria: chromosomal disorders or serious congenital malformations at discharge that would affect growth and development	Intervention: 35 Control: 34	Preterm formula (protein 2.3 g/100 mL, fat 4.1 g/100 mL, carbohydrate 8.5 g/100 mL, energy 80 kcal/100 mL)	Term formula (protein 1.6 g/100 mL, fat 3.5 g/100 mL, carbohydrate 7.2 g/100 mL, energy 67 kcal/100 mL)	Started at term, stopped at 6 months’ CA	Bayley II at 18 months’ CA.
Lucas 1989 [28]	UK	Inclusion criteria: preterm GA < 37 weeks and BW < 1,850 g Exclusion criteria: major congenital abnormality known to impair growth or development, or died before randomization within the first 48 hours	(1) Lucas 1989a: Intervention: 76 Control: 83 (2) Lucas 1989b: Intervention: 173 Control: 170 (3) Lucas 1989c: combined Lucas 1989a and Lucas 1989b: Intervention: 249 Control: 253	(1) Lucas 1989a Preterm formula as sole diet (protein 2.0 g/100 mL, fat 4.9 g/100 mL, carbohydrate 7.0 g/100 mL, energy 80 kcal/100 mL) (2) Lucas 1989b Preterm formula as supplement (3) Lucas 1989c: combined Lucas 1989a and Lucas 1989b	(1) Lucas 1989a: Banked breast milk as sole diet (protein 1.1 g/100 mL, fat 1.7 g/100 mL, carbohydrate 7.1 g/100 mL, energy 46 kcal/100 mL) (2) Lucas 1989b: banked breast milk as supplement (3) Lucas 1989c: combined Lucas 1989a and Lucas 1989b	Started within 48 hours, stopped at discharge or when the infants reached 2,000 g	Bayley II at 9, 18 months’ CA; Blood pressure at 7.5 to 8 years’ and 13 to 16 years’ CA; Triglycerides, HDL, LDL, fasting blood glucose concentration, fasting insulin concentration, insulin resistance (fasting 32–33 split proinsulin) at 13 to 16 years’ CA.
Lucas 1990 [16]	UK	Inclusion criteria: preterm BW < 1,850 g and GA < 37 weeks Exclusion criteria: major congenital abnormality known to impair growth or development or died before randomization within the first 48 hours	(1) Lucas 1990a: Intervention: 81 Control: 79 (2) Lucas 1990b: Intervention: 132 Control: 132 (3) Lucas 1990c: combined Lucas 1990a and Lucas 1990b: Intervention: 213 Control: 211	(1) Lucas 1990a: Preterm formula as sole diet (protein 2.0 g/100 mL, fat 4.9 g/100 mL, carbohydrate 7.0 g/100 mL, energy 80 kcal/100 mL) (2) Lucas 1990b Preterm formula as supplement (3) Lucas 1990c: combined Lucas 1990a and Lucas 1990b	(1) Lucas 1990a: term formula as sole diet (protein 1.5 g/100 mL, fat 3.8 g/100 mL, carbohydrate 7.0 g/100 mL, energy 68 kcal/100 mL) (2) Lucas 1990b: term formula as supplement (3) Lucas 1990c: combined Lucas 1990a and Lucas 1990b	Started within 48 hours, stopped at discharge or when the infants reached 2,000 g	Bayley II at 9, 18 months’ CA; WISC at 7.5 to 8 years’ CA; Blood pressure at 7.5 to 8 years’ and 13 to 16 years’ CA; Triglycerides, HDL, LDL, fasting blood glucose concentration, fasting insulin concentration, insulin resistance (fasting 32–33 split proinsulin) at 13 to 16 years’ CA.
Lucas 1996 [6]	UK	Inclusion criteria: preterm BW < 1,850 g, GA < 37 weeks, and survived to be assigned to a study group between 48 and 72 hours of age Exclusion criteria: major congenital anomalies	Intervention: 137 Control: 138	Fortified human breast milk; fortifier containing protein 0.7 g/100 mL, fat 0.05 g/100 mL, carbohydrate 2.73 g/100 mL, energy 14 kcal/100 mL	Human breast milk	Started within 48 hours, stopped at discharge or when the infants reached 2,000 g	Bayley II at 9, 18 months’ CA.
Lucas 2001 [37]	UK	Inclusion criteria: preterm GA < 37 weeks and BW < 1,750 g Exclusion criteria: congenital malformations or conditions known to affect growth or development	Intervention: 113 Control: 116	Postdischarge formula (protein 1.85 g/100 mL, fat 3.96 g/100 mL, carbohydrate 7.24 g/100 mL, energy 72 kcal/100 mL)	Term formula (protein 1.45 g/100 mL, fat 3.82 g/100 mL carbohydrate 6.96 g/100 mL, energy 68 kcal/100 mL)	Started one week before discharge, stopped at 9 months post term	Bayley II at 18 months’ CA.
Morgan 2014 [29]	UK	Inclusion criteria: preterm GA between 24 to 28 weeks and BW < 1,200 g Exclusion criteria: unlikely to survive the first week after birth; diagnosed with major congenital or chromosomal abnormalities known to affect gastrointestinal function or head growth, including definite parenchymal lesions on cranial ultrasound scan in first 48 hours	Intervention: 74 Control: 76	Standard macronutrient content (parenteral intake with protein 3.8 g/kg/day, fat 3.8 g/kg/day, carbohydrate 15.6 g/kg/day, energy 103 kcal/kg/day)	Higher macronutrient content (parenteral intake with protein 2.8 g/kg/day, fat 2.8 g/kg/day, carbohydrate 13.5 g/kg/day, energy 85 kcal/kg/day)	Started within 120 hours of birth, stopped at 28 days	Bayley III at 2 to 3.5 years of CA.
O’Connor 2008 [38]	Canada	Inclusion criteria: preterm GA < 33 weeks and BW between 750 and 1,800 g who received ≥80% of their total feedings as human milk 3 days before hospital discharge Exclusion criteria: serious congenital or chromosomal anomalies that could affect growth, grade 3 or 4 periventricular or intraventricular hemorrhage, oral steroids within 14 days of randomization, severe asphyxia, and known maternal alcohol or drug abuse	Intervention: 19 Control: 20	Human milk with a multinutrient fortifier (protein 2.0 g/100 mL, fat 4.2 g/100 mL, carbohydrate 8.8 g/100 mL, energy 81 kcal/100 mL)	Unfortified human milk (protein 1.3 g/100 mL, fat 3.9 g/100 mL, carbohydrate 7.2 g/100 mL, energy 68 kcal/100 mL)	Started from discharge, stopped at 12 weeks after discharge	Bayley II at 18 months’ CA.
Roggero 2012 [39]	Italy	Inclusion criteria: preterm GA ≤ 32 weeks or BW ≤ 1,500 g and being fed human milk for 20% of the total milk intake Exclusion criteria: congenital malformations or conditions that interfere with growth or body composition	Intervention: 110 Control: 107	Nutrient-enriched formula (protein 2.0 g/100 mL, fat 4.1 g/100 mL, carbohydrate 7.5 g/100 mL, energy 75 kcal/100 mL)	Term formula (protein 1.4 g/100 mL, fat 3.7 g/100 mL, carbohydrate 7.4 g/100 mL, energy 68 kcal/100 mL)	Started from term CA, stopped at 6 months	GMDS at 24 months’ CA.
Svenningsen 1982 [41]	Sweden	Inclusion criteria: Very low BW preterm with mean BW 1,385 ± 343 g and GA 30.8 ± 2.9 weeks	Intervention: 16 Control: 14	Nutrition enriched formula (protein 2.1 g/100 mL, energy 69.5 kcal/100 mL)	Standard formula (protein 1.6 g/100 mL, energy 68.5 kcal/100 mL)	Started from the third week after birth, stopped at the seventh week after birth	Development impairments at 6 months, 1 and 2 years of age.
Tan 2008 [30]	UK	Inclusion criteria: preterm GA < 29 weeks Exclusion criteria: triplets and higher multiplicity, admitted after 7 days of age, major congenital abnormalities	Intervention: 68 Control: 74	Parenteral intake with protein 4 g/kg/day, fat 4 g/kg/day, carbohydrate 16.3 g/kg/day, energy 117 kcal/kg/day; enteral intake breast milk or formula with target protein 4 g/kg/day, energy 133–150 kcal/kg/day	Parenteral intake with protein 3 g/kg/day, fat 3 g/kg/day, carbohydrate 13.5 g/kg/day, energy 93 kcal/kg/day; enteral intake breast milk or formula with target protein 3.3 g/kg/day, energy 133 kcal/kg/day	Started when infants received parenteral and enteral nutrition from the first week, stopped at 34 weeks’ PMA	Bayley II at 3 and 9 months’ CA.
Zachariassen 2001 [40]	Denmark	Inclusion criteria: preterm GA ≤ 32 weeks, breastfeeding Exclusion criteria: severe diseases or circumstances influencing eating and feeding ability at discharge	Intervention: 105 Control: 102	Fortified mother's milk (protein 1.375 g/day, energy 17.5 kcal/day)	Unfortified mother's milk	Started from shortly before discharge, stopped at 4 months’ CA	Obesity, type 2 diabetes, high blood pressure, blood pressure, triglycerides, HDL, LDL, fasting blood glucose concentration, fasting insulin concentration, at 6 years’ CA.

GMDS measures locomotor skills, personal-social, hearing, language, eye and hand coordination, performance, and practical reasoning. DASI I measures mental and motor development. Bayley-II measures mental and psychomotor development. Bayley-III measures cognitive, motor and language development. The Stanford-Binet test measures cognitive development, including fluid reasoning, knowledge, quantitative reasoning, visual-spatial processing, and working memory.

**Abbreviations:** Bayley-II, Bayley Scales of Infant and Toddler Development-Edition II; Bayley-III, Bayley Scales of Infant and Toddler Development-Edition III; BW, birth weight; CA, corrected age; DASII, Developmental Assessment Scales for Indian Infants-Edition II; GA, gestational age; GMDS, Griffith Mental Developmental Scale; HOMA-IR: Homeostatic Model Assessment of Insulin Resistance; PMA, post-menstrual age; SD, standard deviation; WISC, Wechsler Intelligence Scale for Children

### Risk of bias in included studies

Included studies were of variable methodological quality (S1A and S1B Fig), with 70% having a high risk of attribution bias due to loss to follow-up and 25% at high risk of performance bias because of lack of blinding [6,16,28,30,34]. The high risk of other bias in several studies was because of imbalance of baseline characteristics [22,24,34,36] and different baseline characteristics in each publication [33]. Nearly 30% were at unclear risk of other bias, particularly those supported by formula or fortifier companies where the role of the funders was not clear [6,16,22,27,28,32,33,37]. One study [25] was at high risk of selection bias because the infants were randomised according to the last digit of the infants’ hospital number.

### Co-primary outcome: Cognitive impairment and metabolic risk

**Fig 2:**
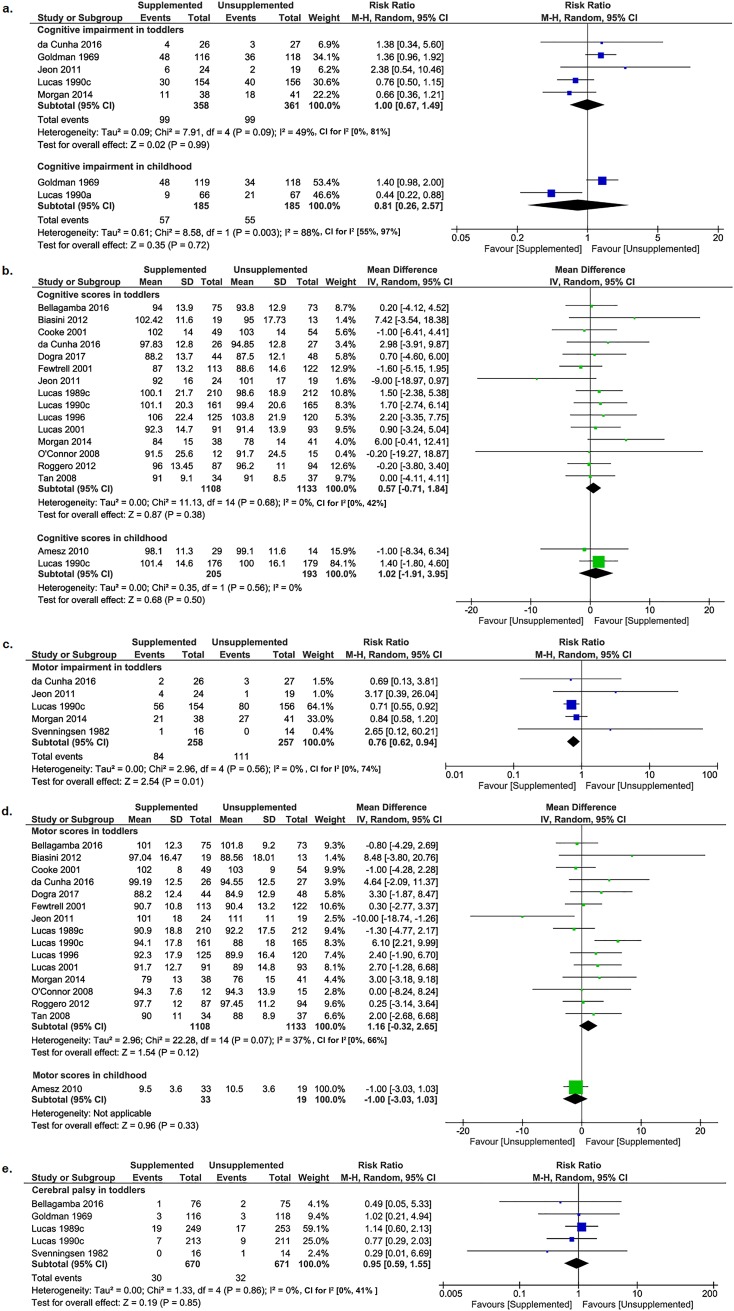
Forest plots of effect of macronutrient supplementation on primary and secondary developmental outcomes. (a) Cognitive impairment (primary outcome); (b) cognitive scores; (c) motor impairment; (d) motor scores; (e) cerebral palsy (all secondary outcomes). Blue boxes in the forest plots represent the dichotomous data; green boxes represent the continuous data. CI, confidence interval; M-H, Mantel-Haenszel; IV, inverse variance.

There was no difference between supplemented and unsupplemented groups in cognitive impairment in toddlers (5 trials [16,27,29,34,36]; 719 children; RR 1.00; 95% CI 0.67–1.49; *P* = 0.99; Fig 2A) or in childhood (2 trials [16,27]; 370 children; RR 0.81; 95% CI 0.26–2.57; *P* = 0.72; Fig 2A).

One trial [40] reported the incidence of obesity, high blood pressure, and type 2 diabetes in childhood, but it was not possible to extract data about the number of individual children who experienced any of these outcomes.

### Secondary developmental outcomes

There was no difference between supplemented and unsupplemented groups in cognitive scores in toddlers (15 trials [6,16,22–24,26,28–30,33,34,36–39]; 2,241 children; MD 0.57; 95% CI −0.71 to 1.84; *P* = 0.38; Fig 2B) or in childhood (2 trials [16,32]; 398 children; MD 1.02; 95% CI −1.91 to 3.95; *P* = 0.50; Fig 2B). Sensitivity analysis including only studies at low risk of bias did not alter the findings of cognitive scores in toddlers (6 trials [16,22,26,28–30]; 1,225 children; MD 0.73; 95% CI −1.05 to 2.51; *P* = 0.42; S2A Fig), and funnel plots (S3A Fig) did not suggest significant bias due to small study effects.

Toddlers in the supplemented group had a lower risk of motor impairment than the unsupplemented group (5 trials [16,29,34,36,41]; 515 children; RR 0.76; 95% CI 0.62 to 0.94; *P* = 0.01; Fig 2C). There was no difference between supplemented and unsupplemented groups in motor scores in toddlers (15 trials [6,16,22–24,26,28–30,33,34,36–39]; 2,241 children; MD 1.16; 95% CI −0.32 to 2.65; *P* = 0.12; Fig 2D) or in childhood (1 trial [32]; 52 children; MD −1.00; 95% CI −3.03 to 1.03; *P* = 0.33; Fig 2D). Sensitivity analysis including only studies at low risk of bias did not alter the findings of motor scores in toddlers (6 trials [16,22,26,28–30], 1,225 children, MD 1.96; 95% CI −0.36 to 4.28; *P* = 0.10; S2B Fig), and funnel plots (S3B Fig) did not suggest significant bias due to small study effects.

There was no clear difference in the incidence of cerebral palsy in toddlers between supplemented and unsupplemented groups (5 trials [16,23,27,28,41]; 1,341 children; RR 0.95; 95% CI 0.59 to 1.55; *P* = 0.85; Fig 2E).

One trial [27] (234 children) reported visual and hearing impairment in toddlers. There was no difference between supplemented and unsupplemented groups in visual impairment (RR 1.02; 95% CI 0.06 to 16.07; *P* = 0.99; S4A Fig) or hearing impairment (RR 0.20; 95% CI 0.01 to 4.19; *P* = 0.30; S4B Fig).

### Secondary metabolic outcomes

**Fig 3:**
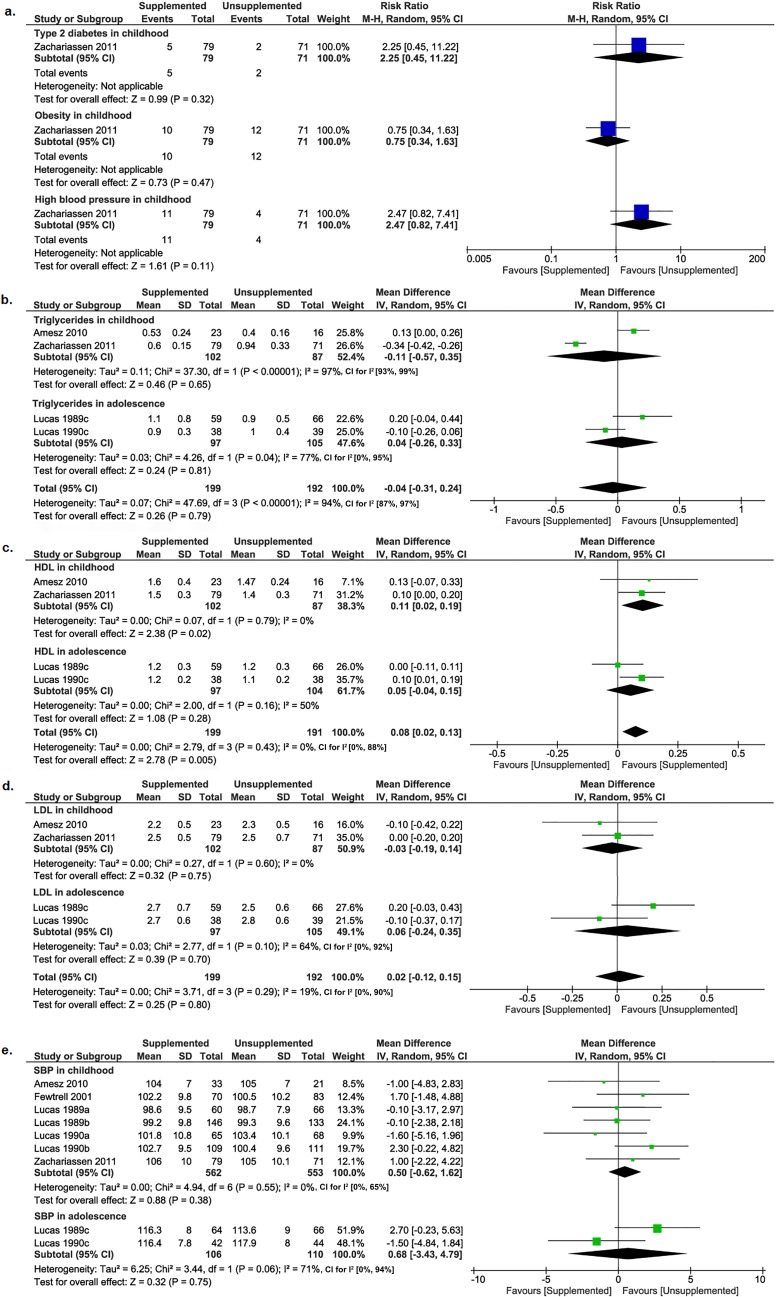
Forest plots of the effect of macronutrient supplements on secondary metabolic outcomes. (a) metabolic risks, (b) triglyceride concentrations, (c) HDL concentrations, (d) LDL concentrations, (e) SBP. Blue boxes in the forest plots represent the dichotomous data; green boxes represent the continuous data. CI, confidence interval; HDL, high-density lipoprotein; IV, inverse variance; LDL, low-density lipoprotein; M-H, Mantel-Haensel; SBP, systolic blood pressure; SD, standard deviation.

One trial (150 children) [40] found no differences between supplemented and unsupplemented groups in childhood for type 2 diabetes (RR 2.25; 95% CI 0.45–11.22; *P* = 0.32), obesity (RR 0.75; 95% CI 0.34–1.63; *P* = 0.47), and high blood pressure (RR 2.47; 95% CI 0.82–7.41; *P* = 0.11; Fig 3A).

There were no differences between supplemented and unsupplemented groups in triglyceride concentrations in childhood (2 trials [32,40]; 189 children; MD −0.11 mmol/L; 95% CI −0.57 to 0.35 mmol/L; *P* = 0.65; Fig 3B) or in adolescence (2 trials [16,28]; 202 children; MD 0.04 mmol/L; 95% CI −0.26 to 0.33 mmol/L; *P* = 0.81; Fig 3B) or at >3 years (4 trials [16,28,32,40]; 391 children; MD −0.04 mmol/L; 95% CI −0.31 to 0.24 mmol/L; *P* = 0.79; Fig 3B).

In childhood, supplemented children had higher HDL concentrations than unsupplemented children (2 trials [32,40]; 189 children; MD 0.11 mmol/L; 95% CI 0.02–0.19 mmol/L; *P* = 0.02; Fig 3C). In adolescence, there was no difference in HDL concentrations between supplemented and unsupplemented groups (2 trials [16,28]; 201 children; MD 0.05 mmol/L; 95% CI −0.04 to 0.15 mmol/L; *P* = 0.28; Fig 3C). At >3 years, supplemented children had higher HDL concentrations than unsupplemented children (4 trials [16,28,32,40]; 391 children; MD 0.08 mmol/L; 95% CI 0.02–0.13 mmol/L; *P* = 0.005; Fig 3C).

There was no difference between supplemented and unsupplemented groups in LDL concentrations in childhood (2 trials [32,40]; 189 children; MD −0.03 mmol/L; 95% CI −0.19 to 0.14 mmol/L; *P* = 0.75; Fig 3D) or in adolescence (2 trials [16,28]; 202 children; MD 0.06 mmol/L; 95% CI −0.24 to 0.35 mmol/L; *P* = 0.70; Fig 3D) or at >3 years (4 trials [16,28,32,40]; 391 children; MD 0.02 mmol/L; 95% CI −0.12 to 0.15 mmol/L; *P* = 0.80; Fig 3D).

There was no difference between supplemented and unsupplemented groups for BMI in childhood (7 trials [16,22,28,32,40]; 1,136 children; MD −0.10 kg/m^2^, 95% CI −0.37 to 0.16 kg/m^2^; *P* = 0.45; S5A Fig) or in adolescence (2 trials [16,28]; 216 children; MD −0.48 kg/m^2^, 95% CI −2.05 to 1.08 kg/m^2^; *P* = 0.55; S5A Fig).

In childhood, supplemented children had lower fasting blood glucose concentrations than unsupplemented children (2 trials [32,40]; 189 children; MD −0.20 mmol/L; 95% CI −0.34 to −0.06 mmol/L; *P* = 0.005; S5B Fig). There was no clear difference between supplemented and unsupplemented groups in fasting blood glucose concentrations in adolescence (2 trials [16,28]; 216 children; MD −0.06 mmol/L; 95% CI −0.25 to 0.12 mmol/L; *P* = 0.49; S5B) or at >3 years (4 trials [16,28,32,40]; 405 children; MD −0.13 mmol/L, 95% CI −0.26 to 0.00 mmol/L; *P* = 0.05; S5B Fig).

There was no clear difference between supplemented and unsupplemented groups in fasting insulin concentrations in childhood (2 trials [32,40]; 189 children; MD 4.38 pmol/L; 95% CI −1.70 to 10.47 pmol/L; *P* = 0.16; S5C Fig), in adolescence (2 trials [16,28]; 216 children; MD −1.18 pmol/L; 95% CI −8.26 to 5.90 pmol/L; *P* = 0.74; S5C Fig), or at >3 years (4 trials [16,28,32,40]; 405 children; MD 2.02 pmol/L; 95% CI −2.59 to 6.64 pmol/L; *P* = 0.39; S5C Fig).

There was no clear difference between supplemented and unsupplemented groups in insulin resistance measured by Homeostatic Model Assessment of Insulin Resistance (HOMA-IR) in childhood (1 trial; 39 children; standard mean difference [SMD] −0.13 pmol/L; 95% CI −0.77 to 0.51 pmol/L; *P* = 0.69; S5D Fig) or measured by fasting 32–33 split proinsulin in adolescence (2 trials [16,28]; 216 children; SMD −0.02 pmol/L; 95% CI −0.71 to 0.68 pmol/L; *P* = 0.96; S5D Fig) or at >3 years (3 trials [16,28,32]; 255 children; SMD −0.04 pmol/L; 95% CI −0.52 to 0.44 pmol/L; *P* = 0.86; S5D Fig).

There were no differences between supplemented and unsupplemented groups for SBP in childhood (7 trials [16,22,28,32,40]; 1,115 children; MD 0.50 mmHg; 95% CI −0.62 to 1.62 mmHg; *P* = 0.38; Fig 3E) or in adolescence (2 trials [16,28]; 216 children; MD 0.68 mmHg; 95% CI −3.43 to 4.79 mmHg; *P* = 0.75; Fig 3E); for diastolic blood pressure (DBP) in childhood (7 trials [16,22,28,32,40]; 1,115 children; MD 0.47 mmHg; 95% CI −0.45 to 1.39 mmHg; *P* = 0.32; S6A Fig) or in adolescence (2 trials [16,28]; 216 children; MD 1.78 mmHg; 95% CI −1.05 to 4.61 mmHg; *P* = 0.22; S6A Fig); or for mean arterial pressure (MAP) in childhood (3 trials [22,32,40]; 357 children; MD 0.95 mmHg; 95% CI −1.19 to 3.09 mmHg; *P* = 0.39; S6B Fig) or in adolescence (2 trials [16,28]; 216 children; MD 1.66 mmHg, 95% CI −3.44 to 6.75 mmHg; *P* = 0.52; S6B Fig).

### Secondary health outcomes

Supplemented and unsupplemented toddlers were not different in the incidence of asthma (3 trials [16,27,28]; 1,011 children; RR 1.02; 95% CI 0.82–1.27; *P* = 0.85; S7A Fig) or eczema (2 trials [16,28]; 777 children; RR 0.96; 95% CI 0.72–1.28; *P* = 0.80; S7B Fig). None of the included trials reported other secondary health outcomes.

### Subgroup analyses

#### Sex

In toddlers, there was no significant sex interaction for cognitive impairment [27]. However, supplemented boys had higher cognitive scores than unsupplemented boys (MD 5.60; 95% CI 1.07–10.14; *P* = 0.02), but there were no differences in girls [16,33] (*P* = 0.03 for interaction). There were no significant sex interactions for motor scores in toddlers [16,33] or SBP in childhood [16,28] (Table 2).

**Table 2:** Summary of subgroup analyses.

	Subgroup	No. of participants (studies)	RR or MD (95% CI)	*P* for overall effect	*I*^2^	*P* for subgroup interaction
**Sex of infants**						
**Cognitive impairment in toddlers**	Boys	116 (1 RCT) [27]	RR = 1.12 (0.74 to 1.70)	0.59	NA	0.19
	Girls	118 (1 RCT) [27]	RR = 1.83 (1.00 to 3.35)	0.05	NA	
**Cognitive scores in toddlers**	Boys	201 (2 RCTs) [16,33]	MD = 5.60 (1.07 to 10.14)	0.02	0%	0.03
	Girls	212 (2 RCTs) [16,33]	MD = −2.04 (−7.04 to 2.95)	0.42	0%	
**Motor scores in toddlers**	Boys	201 (2 RCTs) [16,33]	MD = 4.32 (−4.40 to 13.04)	0.33	88%	0.55
	Girls	212 (2 RCTs) [16,33]	MD = 1.04 (−5.13 to 7.21)	0.74	65%	
**SBP in childhood**	Boys	366 (4 RCTs) [16,28]	MD = 0.70 (−1.88 to 3.28) mmHg	0.59	36%	0.74
	Girls	382 (4 RCTs) [16,28]	MD = 0.08 (−2.46 to 2.62) mmHg	0.95	41%	
**SGA infants**						
**Cognitive scores in toddlers**	SGA	569 (5 RCTs) [16,22,24,28,39]	MD = −0.47 (−5.20 to 4.25)	0.84	65%	NA
**Motor scores in toddlers**	SGA	569 (5 RCTs) [16,22,24,28,39]	MD = 2.70 (−2.02 to 7.42)	0.26	70%	NA
**SBP in childhood**	SGA	267 (4 RCTs) [16,28]	MD = 0.53 (−3.05 to 4.11) mmHg	0.77	49%	NA
**Timing of the supplements**						
**Cognitive impairment in toddlers**	Started in the hospital	623 (3 RCTs) [16,27,29]	RR = 0.91 (0.58 to 1.45)	0.70	69%	0.24
	Across in-hospital and postdischarge periods	None				
	Started after hospital discharge	96 (2 RCTs) [34,36]	RR = 1.78 (0.65 to 4.93)	0.26	0%	
**Motor impairment in toddlers**	Started in the hospital	389 (2 RCTs) [16,29]	RR = 0.75 (0.61 to 0.93)	0.008	0%	0.57
	Across in-hospital and postdischarge periods	30 (1 RCT) [41]	RR = 2.65 (0.12 to 60.21)	0.54	NA	
	Started after hospital discharge	96 (2 RCTs[34,36]	RR = 1.30 (0.30 to 5.70)	0.73	18%	
**Cognitive scores in toddlers**	Started in the hospital	1415 (8 RCTs) [6,16,23,24,26,28–30]	MD = 1.51 (−0.23 to 3.25)	0.09	0%	0.23
	Across in-hospital and postdischarge periods	235 (1 RCTs) [22]	MD = −1.60 (−5.15 to 1.95)	0.38	NA	
	Started after hospital discharge	591 (6 RCTs) [33,34,36–39]	MD = −0.12 (−2.34 to 2.09)	0.91	0%	
**Motor scores in toddlers**	Started in the hospital	1415 (8 RCTs) [6,16,23,24,26,28–30]	MD = 2.04 (−0.08 to 4.16)	0.06	40%	0.48
	Across in-hospital and postdischarge periods	235 (1 RCT) [22]	MD = 0.30 (−2.77 to 3.37)	0.85	NA	
	Started after hospital discharge	591 (6 RCTs) [33,34,36–39]	MD = 0.15 (−2.55 to 2.86)	0.91	44%	
**SBP in childhood**	Started in the hospital	758 (4 RCTs) [16,28]	MD = 0.35 (−1.18 to 1.88) mmHg	0.66	18%	0.72
	Across in-hospital and postdischarge periods	153 (1 RCT) [22]	MD = 1.70 (−1.48 to 4.88) mmHg	0.29	0%	
	Started after hospital discharge	204 (2 RCTs) [32,40]	MD = 0.17 (−2.29 to 2.64) mmHg	0.89	0%	

**Abbreviations:** CI, confidence interval; MD, mean difference; NA, not applicable; RCT, randomised controlled trial; RR, relative risk; SBP, systolic blood pressure.

#### SGA infants

In children born SGA, there were no clear differences between supplemented and unsupplemented groups in cognitive scores in toddlers [16,22,24,28,39], motor scores in toddlers [16,22,24,28,39], or SBP in childhood [16,28] (Table 2).

#### Timing of the supplement

There were no clear differences between supplemented and unsupplemented groups in cognitive impairment in toddlers, cognitive scores in toddlers, motor scores in toddlers, and SBP in the different timing subgroups, and there was no evidence of an interaction between timing and effects of supplements on cognitive impairment in toddlers [16,27,29,34,36], motor impairment in toddlers [16,29,34,36,41], cognitive scores in toddlers [6,16,22–24,26,28–30,33,34,36–39], motor scores in toddlers [6,16,22–24,26,28–30,33,34,36–39], and SBP in childhood [16,22,28,32,40] (Table 2). Toddlers who had received supplements had a lower risk of motor impairment than the unsupplemented groups (RR 0.75; 95% CI 0.61–0.93; *P* = 0.008) if they received the supplements in hospital, but not if they received supplements both in-hospital and post discharge, or only post discharge (Table 2).

Due to insufficient data, we were unable to undertake other preplanned subgroup analyses (S2 Appendix).

### Studies not included in quantitative synthesis

Agosti 2003 [31] reported no difference in overall Griffiths Mental Development Status (GMDS) scores at 12 months between supplemented (mean score = 101) and unsupplemented groups (mean score = 102). In subgroup analyses, supplemented SGA children had better GMDS scores than unsupplemented at 6 months (mean scores 101 versus 95), but the differences did not persist at 9 and 12 months. Supplemented boys also had better GMDS scores than unsupplemented at 6 months (mean scores 102 versus 98) and 9 months (mean scores 106 versus 103) but not 12 months, whereas there was no difference in GMDS scores in supplemented and unsupplemented girls at each age.

Cooper 1988 [25] reported no difference in the overall GMDS scores between supplemented and unsupplemented toddlers (MD 5; 95% CI −21.83 to 11.83; *P* = 0.56).

Friel 1993 [35] reported no difference in GMDS at 12 months between supplemented and unsupplemented groups (mean score 92 versus 90).

### Quality of evidence (GRADE)

There were no data for the outcomes: composite of survival free of any disability, school performance, elevated fasting plasma glucose concentrations at >3 years, and insulin resistance at >3 years. The quality of the evidence was assessed as low or very low for all other development and metabolic outcomes (Table 3).

**Table 3:** GRADE table: Summary of findings.

Supplemented compared to unsupplemented nutrition for children born preterm or SGA					
**Patient or population**: Children born preterm or SGA					
**Setting**: Hospital or NICU					
**Intervention**: Supplemented nutrition					
**Comparison**: Unsupplemented nutrition					
	Anticipated absolute effects* (95% CI)				
Outcomes	**Risk with unsupplemented nutrition**	**Risk with supplemented nutrition**	Relative effect (95% CI)	Number of participants (studies)	Certainty of the evidence (GRADE)
**a. Summary of findings for the developmental outcomes**					
Cognitive impairment in toddlers (primary outcome)	274 per 1,000	**274 per 1,000** (184 to 409)	**RR 1.00** (0.67 to 1.49)	719 (5 RCTs)	⨁◯◯◯ VERY LOW ^a^^,^^b^^,^^c^^,^^d^
Cognitive scores in toddlers	Comparator	Mean cognitive score in the intervention group was 0.57 points higher (0.71 lower to 1.84 higher)		2,241 (15 RCTs)	⨁⨁◯◯ LOW ^a^^,^^b^^,^^d^
Motor impairment in toddlers	432 per 1,000	**328 per 1,000** (268 to 406)	**RR 0.76** (0.62 to 0.94)	515 (5 RCTs)	⨁◯◯◯ VERY LOW ^a^^,^^b^^,^^d^^,^^e^
Motor scores in toddlers	Comparator	Mean motor score in the intervention group was 1.16 points higher (0.32 lower to 2.65 higher)		2,241 (15 RCTs)	⨁⨁◯◯ LOW ^a^^,^^b^^,^^d^
Cerebral palsy in toddlers	48 per 1,000	**45 per 1,000** (28 to 74)	**RR 0.95** (0.59 to 1.55)	1,341 (4 RCTs)	⨁⨁◯◯ LOW ^c^^,^^d^
**b. Summary of findings for the metabolic outcomes**					
Overweight/obesity at >3 years	169 per 1,000	127 per 1,000 (57 to 275)	RR 0.75 (0.34 to 1.63)	150 (1 RCT)	⨁⨁◯◯ LOW ^a^^,^^c^^,^^f^
Triglyceride at >3 years (mmol/L)	Comparator	The mean triglyceride concentration in the intervention group was 0.04 mmol/L lower (0.31 lower to 0.24 higher)		391 (4 RCTs)	⨁◯◯◯ VERY LOW ^d^^,^^g^^,^^h^
HDL at >3 years (mmol/L)	Comparator	The mean HDL concentration in the intervention group was 0.08 mmol/L higher (0.02 higher to 0.13 higher)		390 (4 RCTs)	⨁⨁◯◯ LOW ^d^^,^^g^
LDL at >3 years (mmol/L)	Comparator	The mean LDL concentration in the intervention group was 0.02 mmol/L higher (0.12 lower to 0.15 higher)		391 (4 RCTs)	⨁⨁◯ LOW ^d^^,^^g^
SBP at >3 years (mmHg)	Comparator	The mean SBP in the intervention group was 0.5 mmHg higher (0.62 lower to 1.62 higher)		1,115 (7 RCTs)	⨁⨁◯ LOW ^d^^,^^g^

GRADE Working Group grades of evidence are as follows. High certainty: We are very confident that the true effect lies close to that of the estimate of the effect. Moderate certainty: We are moderately confident in the effect estimate: the true effect is likely to be close to the estimate of the effect, but there is a possibility that it is substantially different. Low certainty: Our confidence in the effect estimate is limited: the true effect may be substantially different from the estimate of the effect. Very low certainty: We have very little confidence in the effect estimate: the true effect is likely to be substantially different from the estimate of effect.

^a^Uncertainty about methods used to generate a random sequence, conceal allocation or blind outcome assessors in some studies.

^b^Baseline characteristics were not balanced in some studies.

^c^CI includes both possible benefit and no benefits from supplementation.

^d^Some of the studies were supported by formula or fortifier companies whose role was not specified.

^e^One study was at high risk of selective reporting bias (infants with cerebral palsy were not included).

^f^Relatively few studies with few participants.

^g^Large losses to follow-up in childhood or beyond.

^h^Substantial heterogeneity existed.

*The risk in the intervention group (and its 95% CI) is based on the assumed risk in the comparison group and the relative effect of the intervention (and its 95% CI).

**Abbreviations:** CI, confidence interval; GRADE, Grading of Recommendations Assessment, Development and Evaluation; HDL, high-density lipoprotein; LDL, low-density lipoprotein; MD, mean difference; NICU, neonatal intensive care unit; RCT, randomised controlled trial; RR, relative risk; SBP, systolic blood pressure; SGA, small for gestational age

## Discussion

We hypothesised that early macronutrient supplements of infants born small would benefit early cognition, but this may be at the cost of worse later metabolic outcomes. In our systematic review and meta-analysis of 21 RCTs and 1 quasi-RCT involving 3,680 infants, we found no evidence that early macronutrient supplementation led to significant changes in cognitive function in children born preterm or SGA. This finding from randomised trials is in contrast to previous observational studies [42,43] that suggest a positive association between macronutrient intake and cognitive development in preterm infants. However, we found limited evidence that early macronutrient supplements decreased the risk of motor impairment in toddlers and, contrary to our hypothesis, improved some metabolic outcomes in childhood. Despite the large numbers of trials and infants included, the evidence is limited by the overall low methodological quality, substantial heterogeneity, and few measures of outcomes after 3 years of age.

Our findings that supplementation decreased the risk of motor impairment in toddlers but did not change motor scores appear contraditory. There are several possible reasons for this. Firstly, the mean scores may not reflect children whose scores fall below specified cut-off points, particularly if data are not normally distributed. Secondly, 15 trials with 2,241 toddlers reported motor scores, but only 5 trials with 515 toddlers reported motor impairment, and only 4 of these reported both motor scores and the incidence of motor impairment. In each of these 4 trials, the differences between supplemented and unsupplemented nutrition groups were in the same direction for both impairments and mean scores. However, the overall finding of decreased motor impairment was dominated by one trial [16] (weighted 64%, Fig 2C), although in the analysis of motor scores, this trial was only weighted 8.3%. Furthermore, the finding of decreased motor impairment was limited by very low-quality evidence. Therefore, we would recommend reporting both scores and numbers of impaired children in future studies.

The effects of macronutrient supplements on developmental outcomes in childhood or later were unclear. Only 1 trial [27] reported cognitive impairment, 2 [32] reported cognitive scores, and 1 [32] reported motor scores in childhood; none reported differences between supplemented and unsupplemented children. This limited evidence suggests that although macronutrient supplements may improve early motor but not cognitive development, these effects may not persist in later life.

Contrary to our hypothesis, we did not find evidence of increased adverse cardiometabolic risk factors after early macronutrient supplementation; rather, we found that children in the supplemented groups had higher HDL concentrations in childhood. Others [44,45] have reported an inverse association between HDL cholesterol concentrations in childhood and cardiometabolic risks in adulthood. However, mean HDL concentrations in both supplemented and unsupplemented groups in our systematic review were below the 10th percentile (2.2 mmol/L) [46], suggesting that all included children are at increased risk of cardiometabolic disease but that those in the unsupplemented groups might be at greater risk.

We also found that supplemented children had lower fasting blood glucose concentrations than unsupplemented children. Childhood fasting blood glucose concentrations are inversely related to pre-diabetes and diabetes in adulthood, especially if the glucose concentration is above 4.7 mmol/L [47]. The mean fasting blood concentrations in studies included in this review were close to or above this threshold, again suggesting that both groups may be at increased risk of later diabetes but that the unsupplemented group might be at greater risk. We also did not detect differences in other metabolic risk factors or blood pressure between supplemented and unsupplemented groups in childhood or in adolescence. Thus, the evidence from this review of randomised trials suggests that, contrary to findings from observational studies [13,44,45], early macronutrient supplementation of preterm and SGA infants does not have adverse effects on later metabolic outcomes.

Interpretation of these findings is limited by heterogeneity and the small number of trials reporting longer-term outcomes. Some of the heterogeneity may be due to the different types of interventions. For example, for the analysis of triglyceride concentrations in childhood, infants in one trial were fed formula [32] and in the other fed breast milk [40] as the main diet. Similarly, for the analysis of metabolic outcomes and blood pressure in adolescence, one trial compared preterm formula with banked breast milk as sole diet or supplement, while the other compared preterm formula with term formula [16]. Because breastfeeding itself has been associated with lower later blood pressure and risk of obesity [48–50], this may contribute to the heterogeneity in these results, although the breastfeeding studies are also observational and potentially confounded by the social determinants of these health outcomes.

In the subgroup analyses, based on limited data, we found that in toddlers, supplemented compared with unsupplemented boys had no difference in the incidence of cognitive impairment, but had a 5.6-point advantage on cognitive scores (95% CI 1.07–10.14). However, there was no difference in cognitive scores between supplemented and unsupplemented girls. A sex-specific response to early nutrient supplements has also been reported in animal studies [51,52]. However, few studies of nutritional supplements in infants born small have reported outcomes separately for boys and girls. A planned individual participant data (IPD) meta-analysis (PROSPERO CRD42017072683) may prove helpful to further explore possible sex differences in the effects of macronutrient supplements in human infants born small.

In the subgroup of infants born SGA, there appeared to be no effect of supplements on cognitive and motor scores in toddlers,or SBP in childhood. However, only 5 trials reported this subgroup separately, and there was substantial heterogeneity. In one trial [24], the unsupplemented group was of higher birth weight and gestational age than the supplemented group, although heterogeneity was still substantial after exclusion of this trial.

In the subgroup analysis of different timing of supplements, there was again no difference in cognitive impairment, but toddlers in the supplemented group had better motor development only when the intervention was given in hospital. Furthermore, timing of supplements may have contributed to the substantial heterogeneity in the subgroup analyses of motor scores of boys and girls separately. Boys who received supplemented nutrition during initial hospitalisation had better motor scores than unsupplemented boys [16], but those who received supplemented nutrition after hospital discharge did not [33]. The in-hospital period aligns with the third trimester of gestation, when there is extensive fetal brain development, and the brain accounts for 60% of total oxygen and caloric consumption. Adequate nutrients are therefore most likely to be important to support brain development during this critical period [53,54]. These findings in one subgroup must be interpreted with caution but may suggest that providing preterm and SGA infants with supplemented nutrition during initial hospitalisation rather than later is more likely to benefit later developmental outcomes.

Three previous systematic reviews have compared the effect of supplemented versus unsupplemented formula started after hospital discharge, fortified versus unfortified breastmilk started in hospital or after hospital discharge [11,17,18]. Each identified a different single eligible trial reporting developmental outcomes [6,37,38]. All 3 trials reported no differences in cognitive and motor scores at 18 months, and none reported long-term metabolic or developmental outcomes after this age. Our study included all eligible trials regardless of type and timing of intervention and included all 3 of the previously reported trials plus another 19 trials, allowing more extensive analysis of some long-term outcomes.

There were some limitations to our study. The quality of evidence was low in many trials. Most were conducted more than 20 years ago, and the findings were not reported according to current guidelines. In particular, methodological details, including blinding of outcome assessment and the role of commercial sponsors, were unclear. Most trials were at high risk of attrition bias, which may introduce bias and loss of power [55] so that there can be limited confidence in the effect estimates. However, in the sensitivity analysis for cognitive and motor scores including only high-quality trials, the results were in the same direction, suggesting that this may not be major source of bias. There was a wide variety of interventions used, including different timing, type, duration, and routes of supplementation and substantial heterogeneity of findings. In addition, some studies reported multiple outcomes, and some research teams reported several different studies, which may result in a lack of independence that is not accounted for in our analyses. Furthermore, we analysed multiple outcomes, multiple time points, and a large number of subgroups, which increases the risk of type 1 error [56], and the subgroup findings in particular should be interpreted with caution.

Although 22 trials have been undertaken involving >3,000 infants, data regarding the effects of early macronutrient supplements on long-term developmental and metabolic outcomes are limited. In addition to new trials, longer-term follow-up of previous trials would provide critical evidence about the effect of macronutrients on long-term developmental and metabolic outcomes of preterm and SGA infants.

Contrary to the findings from observational studies, current low-quality evidence from randomised trials suggests that early macronutrient supplementation of infants born small does not alter later cognition but may decrease motor impairment in toddlers and improve some metabolic outcomes in childhood.

## Supporting information

S1 Checklist PRISMA checklist. PRISMA, Preferred Reporting Items for Systematic Reviews and Meta-Analyses.
(DOC)

S1 Appendix List of outcomes. (DOCX)

S2 Appendix List of planned subgroup analyses. (DOCX)

S3 Appendix List of all references of included studies. (DOCX)

S1 Table Search strategy. (DOCX)

S1 Fig Risk of bias. (a) Risk of bias graph: review authors’ judgements about each risk of bias item presented as percentages across all included studies. (b) Risk bias summary: review authors’ judgements about each risk of bias item for each included study.
(TIF)

S2 Fig Sensitivity analyses. Forest plots of effect of macronutrient supplementation on cognitive scores and motor scores including trials with low risk of bias. (a) Cognitive scores, (b) motor scores.
(TIF)

S3 Fig Funnel plots. Funnel plots of supplemented versus unsupplemented nutrition for the outcomes of cognitive and motor scores in toddlers. (a) Cognitive scores, (b) motor scores. The middle dashed line indicates the overall MD. The dashed lines either side represent the pseudo 95% CIs. MD, mean difference.
(TIF)

S4 Fig Forest plots of the effect of macronutrient supplementation on other developmental outcomes in toddlers. (a) Visual impairment, (b) hearing impairment.
(TIF)

S5 Fig Forest plots of the effects of macronutrient supplementation on other metabolic outcomes. (a) BMI, (b) fasting blood glucose concentrations, (c) fasting insulin concentrations, (d) insulin resistance. BMI, body mass index.
(TIF)

S6 Fig Forest plots of the effects of macronutrient supplementation on blood pressure. (a) DBP, (b) MAP. DBP, diastolic blood pressure; MAP, mean arterial pressure.
(TIF)

S7 Fig Forest plots of the effects of macronutrient supplementation on asthma and eczema in toddlers. (a) Asthma, (b) eczema.
(TIF)

S1 Protocol (DOCX)

## Abbreviations

BMI: body mass index
CA: corrected age
CI: confidence interval
DASII: Developmental Assessment Scales for Indian Infants-Edition II
DBP: diastolic blood pressure
GDT: Guideline Development Tool
GMDS: Griffiths Mental Development Status
GRADE: Grading of Recommendations Assessment, Development and Evaluation
HDL: high-density lipoprotein
HOMA-IR: Homeostatic Model Assessment of Insulin Resistance
IPD: individual participant data
IV: inverse variance
LDL: low-density lipoprotein
MAP: mean arterial pressure
MD: mean difference
M-H: Mantel-Haenszel
PMA: post-menstrual age
PRISMA: Preferred Reporting Items for Systematic Reviews and Meta-Analyses
RCT: randomised controlled trial
RR: relative risk
SBP: systolic blood pressure
SD: standard deviation
SGA: small for gestational age
SMD: standard mean difference
WISC: Wechsler Intelligence Scale for Children

## References

[1] Blencowe H, Cousens S, Chou D, Oestergaard M, Say L, Moller AB, et al Born too soon: the global epidemiology of 15 million preterm births. Reprod Health. 2013;10 Suppl 1:S2. 24625129 10.1186/1742-4755-10-S1-S2 PMC3828585

[2] Scharf RJ, Stroustrup A, Conaway MR, DeBoer MD. Growth and development in children born very low birthweight. Arch Dis Child Fetal Neonatal Ed. 2016;101(5):F433–F8. 10.1136/archdischild-2015-309427 26627552 PMC5494252

[3] Katz J, Lee ACC, Kozuki N, Lawn JE, Cousens S, Blencowe H, et al Mortality risk in preterm and small-for-gestational-age infants in low-income and middle-income countries: a pooled country analysis. Lancet. 2013;382(9890):417–25. 10.1016/S0140-6736(13)60993-9 23746775 PMC3796350

[4] Christian P, Lee SE, Angel MD, Adair LS, Arifeen SE, Ashorn P, et al Risk of childhood undernutrition related to small-for-gestational age and preterm birth in low- and middle-income countries. Int J Epidemiol. 2013;42(5):1340–55. 10.1093/ije/dyt109 23920141 PMC3816349

[5] Mericq V, Martinez-Aguayo A, Uauy R, Iniguez G, Van der Steen M, Hokken-Koelega A. Long-term metabolic risk among children born premature or small for gestational age. Nat Rev Endocrinol. 2017;13(1):50–62. 10.1038/nrendo.2016.127 27539244

[6] Lucas A, Fewtrell MS, Morley R, Lucas PJ, Baker BA, Lister G, et al Randomized outcome trial of human milk fortification and developmental outcome in preterm infants. Am J Clin Nutr. 1996;64(2):142–51. 10.1093/ajcn/64.2.142 8694013

[7] Lucas A, Morley R, Cole TJ. Randomised trial of early diet in preterm babies and later intelligence quotient. BMJ. 1998;317(7171):1481–7. 10.1136/bmj.317.7171.1481 9831573 PMC28727

[8] Lucas A, Morley R, Cole TJ, Gore SM. A randomised multicentre study of human milk versus formula and later development in preterm infants. Arch Dis Child Fetal Neonatal Ed. 1994;70(2):F141–6. 10.1136/fn.70.2.f141 8154907 PMC1061016

[9] Kumar RK, Singhal A, Vaidya U, Banerjee S, Anwar F, Rao S. Optimizing nutrition in preterm low birth weight infants-consensus summary. Front Nutr. 2017;4:20 10.3389/fnut.2017.00020 28603716 PMC5445116

[10] Isaacs EB, Morley R, Lucas A. Early diet and general cognitive outcome at adolescence in children born at or below 30 weeks gestation. J Pediatr. 2009;155(2):229–34. 10.1016/j.jpeds.2009.02.030 19446846

[11] Brown JVE, Embleton ND, Harding JE, McGuire W. Multi-nutrient fortification of human milk for preterm infants. Cochrane Database Syst Rev. 2016(5):CD000343 10.1002/14651858.CD000343.pub3 27155888

[12] Belfort MB, Rifas-Shiman SL, Sullivan T, Collins CT, McPhee AJ, Ryan P, et al Infant growth before and after term:eEffects on neurodevelopment in preterm infants. Pediatrics. 2011;128(4):E899–E906. 10.1542/peds.2011-0282 21949135 PMC3182845

[13] Belfort MB, Gillman MW, Buka SL, Casey PH, McCormick MC. Preterm infant linear growth and adiposity gain: trade-offs for later weight status and intelligence quotient. J Pediatr. 2013;163(6):1564–U71. 10.1016/j.jpeds.2013.06.032 23910982 PMC3834090

[14] Ong KK, Loos RJ. Rapid infancy weight gain and subsequent obesity: systematic reviews and hopeful suggestions. Acta Paediatr. 2006;95(8):904–8. 10.1080/08035250600719754 16882560

[15] Peacock JL, Marston L, Marlow N, Calvert SA, Greenough A. Neonatal and infant outcome in boys and girls born very prematurely. Pediatr Res. 2012;71(3):305–10. 10.1038/pr.2011.50 22258087

[16] Lucas A, Morley R, Cole TJ, Gore SM, Lucas PJ, Crowle P, et al Early diet in preterm babies and developmental status at 18 months. Lancet. 1990;335(8704):1477–81. 10.1016/0140-6736(90)93026-l 1972430

[17] Young L, Embleton ND, McGuire W. Nutrient-enriched formula versus standard formula for preterm infants following hospital discharge. Cochrane Database Syst Rev. 2016(12): CD004696. 27958643 10.1002/14651858.CD004696.pub5 PMC6463855

[18] Young L, Embleton ND, McCormick FM, McGuire W. Multinutrient fortification of human breast milk for preterm infants following hospital discharge. Cochrane Database of Syst Rev. 2013(2):CD004866. 23450556 10.1002/14651858.CD004866.pub4 PMC8855689

[19] Higgins JPT, Green S (editors), The Cochrane Collaboration. Cochrane handbook for systematic reviews of interventions version 5.1.0 [updated March 2011] 2011. Available from: www.cochrane-handbook.org. [cited ].

[20] Schünemann H, Brozek J, Guyatt G, Oxman A, editors. GRADE handbook for grading quality of evidence and strength of recommendations. Updated October 2013. 2013.

[21] Review Manager (RevMan) [Computer program]. Version 5.3. The Nordic Cochrane Centre, The Cochrane Collaboration, 2014: Copenhagen.

[22] Fewtrell MS, Morley R, Abbott RA, Singhal A, Stephenson T, MacFadyen UM, et al Catch-up growth in small-for-gestational-age term infants: a randomized trial. Am J Clin Nutr. 2001;74(4):516–23. 10.1093/ajcn/74.4.516 11566651

[23] Bellagamba MP, Carmenati E, D'Ascenzo R, Malatesta M, Spagnoli C, Biagetti C, et al One extra gram of protein to preterm infants from birth to 1800 g: a single-blinded randomized clinical trial. J Pediatr Gastroenterol Nutr. 2016;62(6):879–84. 10.1097/MPG.0000000000000989 26418211

[24] Biasini A, Marvulli L, Neri E, China M, Stella M, Monti F. Growth and neurological outcome in ELBW preterms fed with human milk and extra-protein supplementation as routine practice: do we need further evidence? J Matern Fetal Neonatal Med. 2012;25 Suppl 4:72–4. 10.3109/14767058.2012.715032 22958024

[25] Cooper PA, Rothberg AD, Davies VA. Three year growth and developmental follow up of very low birthweight infants fed own mother's milk (OMM), a premature infant formula (PF) or one of two standard formulas. Pediatr Res. 1988;23:445A. 10.1097/00005176-198904000-00015 2709266

[26] Dogra S, Thakur A, Garg P, Kler N. Effect of differential enteral protein on growth and nurodevelopment in infants <1500 g: a randomized controlled trial. J Pediatr. 2017;64(5):e126–e32. 10.1097/MPG.0000000000001451 27801753

[27] Goldman HI, Freudenthal R, Holland B, Karelitz S. Clinical effects of two different levels of protein intake on low-birth-weight infants. J Pediatr. 1969;74(6):881–9. 10.1016/s0022-3476(69)80222-2 5781798

[28] Lucas A, Morley R, Cole TJ, Gore SM, Davis JA, Bamford MF, et al Early diet in preterm babies and developmental status in infancy. Arch Dis Child. 1989;64(11):1570–8. 10.1136/adc.64.11.1570 2690739 PMC1792630

[29] Morgan C, McGowan P, Herwitker S, Hart AE, Turner MA. Postnatal head growth in preterm infants: a randomized controlled parenteral nutrition study. Pediatrics. 2014;133(1):e120–8. 10.1542/peds.2013-2207 24379229

[30] Tan MJ, Cooke RW. Improving head growth in very preterm infants—a randomised controlled trial I: neonatal outcomes. Arch Dis Child Fetal Neonatal Ed. 2008;93(5):F337–41. 10.1136/adc.2007.124230 18252814

[31] Agosti M, Vegni C, Calciolari G, Marini A, Group GS. Post-discharge nutrition of the very low-birthweight infant: interim results of the multicentric GAMMA study. Acta Paediatr Suppl. 2003;91(441):39–43. 14599040 10.1111/j.1651-2227.2003.tb00644.x

[32] Amesz EM, Schaafsma A, Cranendonk A, Lafeber HN. Optimal growth and lower fat mass in preterm infants fed a protein-enriched postdischarge formula. J Pediatr Gastroenterol Nutr. 2010;50(2):200–7. 10.1097/MPG.0b013e3181a8150d 19881394

[33] Cooke RJ, Embleton ND, Griffin IJ, Wells JC, McCormick KP. Feeding preterm infants after hospital discharge: growth and development at 18 months of age. Pediatr Res. 2001;49(5):719–22. 10.1203/00006450-200105000-00018 11328958

[34] da Cunha RD, Lamy Filho F, Rafael EV, Lamy ZC, de Queiroz AL. Breast milk supplementation and preterm infant development after hospital discharge: a randomized clinical trial. J Pediatr (Rio J). 2016;92(2):136–42. 10.1016/j.jped.2015.04.004 26403703

[35] Friel JK, Andrews WL, Matthew JD, McKim E, French S, Long DR. Improved growth of very low birthweight infants. Nutr Res. 1993;13(6):611–20.

[36] Jeon GW, Jung YJ, Koh SY, Lee YK, Kim KA, Shin SM, et al Preterm infants fed nutrient-enriched formula until 6 months show improved growth and development. Pediatr Int. 2011;53(5):683–8. 10.1111/j.1442-200X.2011.03332.x 21342352

[37] Lucas A, Fewtrell MS, Morley R, Singhal A, Abbott RA, Isaacs E, et al Randomized trial of nutrient-enriched formula versus standard formula for postdischarge preterm infants. Pediatrics. 2001;108(3):703–11. 10.1542/peds.108.3.703 11533340

[38] O'Connor DL, Khan S, Weishuhn K, Vaughan J, Jefferies A, Campbell DM, et al Growth and nutrient intakes of human milk-fed preterm infants provided with extra energy and nutrients after hospital discharge. Pediatrics. 2008;121(4):766–76. 10.1542/peds.2007-0054 18381542

[39] Roggero P, Gianni ML, Amato O, Liotto N, Morlacchi L, Orsi A, et al Growth and fat-free mass gain in preterm infants after discharge: a randomized controlled trial. Pediatrics. 2012;130(5):e1215–21. 10.1542/peds.2012-1193 23109680

[40] Zachariassen G, Faerk J, Grytter C, Esberg BH, Hjelmborg J, Mortensen S, et al Nutrient enrichment of mother's milk and growth of very preterm infants after hospital discharge. Pediatrics. 2011;127(4):e995–e1003. 10.1542/peds.2010-0723 21402642

[41] Svenningsen NW, Lindroth M, Lindquist B. A comparative study of varying protein intake in low birthweight infant feeding. Acta Paediatr Suppl. 1982;296:28–31. 10.1111/j.1651-2227.1982.tb09590.x 6961737

[42] Cormack BE, Bloomfield FH, Dezoete A, Kuschel CA. Does more protein in the first week of life change outcomes for very low birthweight babies? J Paediatr Child Health. 2011;47(12):898–903. 10.1111/j.1440-1754.2011.02106.x 21658149

[43] Stephens BE, Walden RV, Gargus RA, Tucker R, McKinley L, Mance M, et al First-week protein and energy intakes are associated with 18-month developmental outcomes in extremely low birth weight infants. Pediatrics. 2009;123(5):1337–43. 10.1542/peds.2008-0211 19403500

[44] Mattsson N, Ronnemaa T, Juonala M, Viikari JS, Raitakari OT. Childhood predictors of the metabolic syndrome in adulthood. The cardiovascular risk in young Finns Study. Ann Med. 2008;40(7):542–52. 10.1080/07853890802307709 18728920

[45] Juonala M, Viikari JSA, Kahonen M, Taittonen L, Laitinen T, Hutri-Kahonen N, et al Life-time risk factors and progression of carotid atherosclerosis in young adults: the cardiovascular risk in Young Finns study. Eur Heart J. 2010;31(14):1745–51. 10.1093/eurheartj/ehq141 20501481

[46] McNeal CJ, Underland L, Wilson DP, Blackett PR. Pediatric lipid screening. Clin Lipidol. 2013;8(4):425–36.

[47] Nguyen QM, Srinivasan SR, Xu JH, Chen W, Berenson GS. Fasting plasma glucose levels within the normoglycemic range in childhood as a predictor of prediabetes and type 2 diabetes in adulthood: the Bogalusa Heart Study. Arch Pediatr Adolesc Med. 2010;164(2):124–8. 10.1001/archpediatrics.2009.268 20124140

[48] Martin RM, Gunnell D, Smith GD. Breastfeeding in infancy and blood pressure in later life: systematic review and meta-analysis. Am J Epidemiol. 2005;161(1):15–26. 10.1093/aje/kwh338 15615909

[49] Horta BL, Bahl R, Martines JC, Victora CG, World Health Organization. Evidence on the long-term effects of breastfeeding: systematic reviews and meta-analyses. World Health Organization. 2007.

[50] Uwaezuoke SN, Eneh CI, Ndu IK. Relationship between exclusive breastfeeding and lower risk of childhood obesity: a narrative review of published evidence. Clin Med Insights Pediatr. 2017;11. 10.1177/1179556517690196 PMC5398325 28469518

[51] Amissah E, Lin L, Gamble GD, Crowther CA, Bloomfield FH, Harding JE. Macronutrient supplements in preterm and small-for-gestational-age animals: a systematic review and meta-analysis. Sci Rep. 2019;9(1):14715 10.1038/s41598-019-51295-6 31605011 PMC6789152

[52] Berry MJ, Jaquiery AL, Oliver MH, Harding JE, Bloomfield FH. Neonatal milk supplementation in lambs has persistent effects on growth and metabolic function that differ by sex and gestational age. Br J Nutr. 2016;116(11):1912–25. 10.1017/S0007114516004013 27974050

[53] Georgieff MK, Ramel SE, Cusick SE. Nutritional influences on brain development. Acta Paediatr. 2018;107(8):1310–21. 10.1111/apa.14287 29468731 PMC6045434

[54] Ramel SE, Georgieff MK. Preterm nutrition and the brain. World Rev Nutr Diet. 2014;110:190–200. 10.1159/000358467 24751630

[55] Fewtrell MS, Kennedy K, Singhal A, Martin RM, Ness A, Hadders-Algra M, et al How much loss to follow-up is acceptable in long-term randomised trials and prospective studies? Arch Dis Child. 2008;93(6):458–61. 10.1136/adc.2007.127316 18495909

[56] Bender R, Bunce C, Clarke M, Gates S, Lange S, Pace NL, et al Attention should be given to multiplicity issues in systematic reviews. J Clin Epidemiol. 2008;61(9):857–65. 10.1016/j.jclinepi.2008.03.004 18687287

