# Supplementary material for: Impact of macronutrient supplements for children born preterm or small for gestational age on developmental and metabolic outcomes: A systematic review and meta-analysis
Source: PLoS Med. 2019 Oct 30;16(10):e1002952. doi: 10.1371/journal.pmed.1002952 (PMC6821063; doi:10.1371/journal.pmed.1002952)
Supplement: S1 Appendix — (DOCX) [file pmed.1002952.s002.docx]

**S1 Appendix. List of outcomes**

**The co-primary outcomes were cognitive impairment and any metabolic risk.**

(1) Cognitive impairment: below -1SD on standard tests of cognitive development (toddlers) or cognition/intelligence quotient (later ages) or as defined by trialists.

(2) Metabolic risk: Any of the following (defined by trialists)

- Overweight/obese
- Increased waist circumference
- Increased fat mass or fat mass percentage
- Elevated plasma triglyceride concentrations
- Low high-density lipoprotein (HDL) concentrations
- Elevated low-density lipoprotein (LDL) concentrations
- Elevated fasting plasma glucose concentrations
- Insulin resistance
- Impaired glucose tolerance
- Diagnosis of type 2 diabetes
- High blood pressure
- Impaired flow-mediated vasodilatation

**Secondary outcomes:**

- Composite of survival free of any disability (including death, cerebral palsy, cognition/intelligence delay or impairment, motor development delay or impairment, language delay, visual impairment, hearing impairment)
- Cognition/intelligence delay or impairment (below -1SD on standard tests of cognitive development or as defined by trialists)
- Cognition/intelligence scores
- Motor delay or impairment (below -1SD on standard tests of motor development or as defined by trialists)
- Motor development scores
- Cerebral palsy
- Severity of cerebral palsy
- Language delay
- Language development scores
- Visual impairment
- Hearing impairment
- School performance
- Measures of psychological well-being
- Metabolic outcomes
- Waist circumference
- Overweight/obese
- Ponderal Index
- Body mass index (BMI)
- Type-2 diabetes
- Blood lipid concentrations (triglycerides, HDL, LDL, HDL:LDL)
- Fasting blood glucose concentrations
- Insulin concentrations
- Insulin resistance (HOMA)
- Glucose tolerance
- IGF-1 concentration
- Cardiovascular risk outcomes
- Blood pressure (systolic blood pressure (SBP), diastolic blood pressure (DBP), mean arterial pressure (MAP))
- Flow-mediated vasodilatation
- Measures of sympathetic and parasympathetic tone (e.g. heart rate variability)
- Cardiac size and structure
- Brain development
- Whole brain, white matter and grey matter volumes and volumes of individual brain regions
- Brain maturation measured using MRI (white matter tracts, measures of diffusivity, myelination, surface folding)
- Functional brain imaging
- Health outcomes
- Allergies (eczema, asthma, hayfever),
- Respiratory function
- Hospitalisation (duration, frequency),
- Health care utilisation
- Death: neonatal or later death, up to the time of follow-up and cause of death
- Quality of life
- General health and use of healthcare resources.
- Adverse events.
- Cost
