# Supplementary material for: Impact of macronutrient supplements for children born preterm or small for gestational age on developmental and metabolic outcomes: A systematic review and meta-analysis
Source: PLoS Med. 2019 Oct 30;16(10):e1002952. doi: 10.1371/journal.pmed.1002952 (PMC6821063; doi:10.1371/journal.pmed.1002952)
Supplement: S2 Appendix — (DOCX) [file pmed.1002952.s003.docx]

**S2 Appendix. Planned subgroup analyses**

Where data were available, we planned to conduct subgroup analyses to explore whether the effects of supplements differ between subgroups and test for interaction terms.

1. Sex of infant (male vs female);
2. Size of infant at birth (1kg or less vs more than 1kg at birth);
3. Size for gestation of the infant (10th centile or less vs more than 10th centile);
4. Gestational age of infant at birth (28 completed weeks or less vs 29 to 32 completed weeks vs 33 to 36 weeks)
5. Timing of supplement

In hospital nutrition: the intervention was commenced in hospital or on average ended at 42 weeks’ postmenstrual age or earlier,

Post-discharge nutrition: the intervention was commenced after discharge or on average started at 36 weeks’ postmenstrual age or later,

Both in hospital and post-discharge nutrition: the intervention was commenced in the hospital and continued post-discharge;

1. Type of supplement (protein vs carbohydrate vs fat vs multicomponent and their interactions);
2. Breast milk vs formula as primary milk feed;
3. Duration of supplement (1 to 2 weeks vs 3 to 6 weeks vs more than 7 weeks);
4. Different epochs (conducted up to the year of 2000 vs conducted in or after the year of 2001);
