# Supplementary material for: Impact of macronutrient supplements for children born preterm or small for gestational age on developmental and metabolic outcomes: A systematic review and meta-analysis
Source: PLoS Med. 2019 Oct 30;16(10):e1002952. doi: 10.1371/journal.pmed.1002952 (PMC6821063; doi:10.1371/journal.pmed.1002952)
Supplement: S3 Appendix — (DOCX) [file pmed.1002952.s004.docx]

**S3 Appendix. List of all references of included studies**

**Agosti 2013**

1. Agosti M, Vegni C, Calciolari G, Marini A, Gamma Study Group. Post-discharge nutrition of the very low-birthweight infant: interim results of the multicentric GAMMA study. Acta Paediatr Suppl. 2003;91(441):39-43.

**Amesz 2010**

1. Ruys CA, Broring T, van Schie PEM, van de Lagemaat M, Rotteveel J, Finken MJJ, et al. Neurodevelopment of children born very preterm and/or with a very low birth weight: 8-Year follow-up of a nutritional RCT. Clin Nutr ESPEN. 2019;30:190-8.

2. Ruys CA, van de Lagemaat M, Finken MJ, Lafeber HN. Follow-up of a randomized trial on postdischarge nutrition in preterm-born children at age 8 y. Am J Clin Nutr. 2017;106(2):549-58.

3. van de Lagemaat M, Rotteveel J, Schaafsma A, van Weissenbruch MM, Lafeber HN. Higher vitamin D intake in preterm infants fed an isocaloric, protein- and mineral-enriched postdischarge formula is associated with increased bone accretion. J Nutr. 2013;143(9):1439-44.

4. van de Lagemaat M, Rotteveel J, van Weissenbruch MM, Lafeber HN. Increased gain in bone mineral content of preterm infants fed an isocaloric, protein-, and mineral-enriched postdischarge formula. Eur J Nutr. 2013;52(7):1781-5.

5. van de Lagemaat M, Rotteveel J, Muskiet FA, Schaafsma A, Lafeber HN. Post term dietary-induced changes in DHA and AA status relate to gains in weight, length, and head circumference in preterm infants. Prostaglandins Leukot Essent Fatty Acids. 2011;85(6):311-6.

6. Amesz EM, Schaafsma A, Cranendonk A, Lafeber HN. Optimal growth and lower fat mass in preterm infants fed a protein-enriched postdischarge formula. J Pediatr Gastroenterol Nutr. 2010;50(2):200-7.

7. Amesz. Feeding Normo-Caloric Protein Enriched Formula after Term Does Not Affect Growth but Improves Lean Body Mass Gain in Preterm Infants. EPAS2007. 2007; <http://www.abstracts2view.com/pas/(5740.8>).

8. Amesz. Normocaloric enriched formula after term does not affect growth and body composition in preterm infants. Eur J Pediatr. 2006;165.

**Bellagamba 2016**

1. Bellagamba MP, Carmenati E, D'Ascenzo R, Malatesta M, Spagnoli C, Biagetti C, et al. One extra gram of protein to preterm infants from birth to 1800 g: a single-blinded randomized clinical trial. J Pediatr Gastroenterol Nutr. 2016;62(6):879-84.

**Biasini 2012**

1. Biasini A, Monti F, Laguardia MC, Stella M, Marvulli L, Neri E. High protein intake in human/maternal milk fortification for </=1250 gr infants: intrahospital growth and neurodevelopmental outcome at two years. Acta Biomed. 2018;88(4):470-6.

2. Biasini A, Marvulli L, Neri E, China M, Stella M, Monti F. Growth and neurological outcome in ELBW preterms fed with human milk and extra-protein supplementation as routine practice: do we need further evidence? J Matern Fetal Neonatal Med. 2012;25 Suppl 4:72-4.

**Cooke 2001**

1. Cooke RJ, Embleton ND, Griffin IJ, Wells JC, McCormick KP. Feeding preterm infants after hospital discharge: growth and development at 18 months of age. Pediatr Res. 2001;49(5):719-22.

2. Cooke RJ, Griffin IJ, McCormick K. Adiposity is not altered in preterm infants fed with a nutrient-enriched formula after hospital discharge. Pediatr Res. 2010;67(6):660-4.

3. Cooke RJ, McCormick K, Griffin IJ, Embleton N, Faulkner K, Wells JC, et al. Feeding preterm infants after hospital discharge: effect of diet on body composition. Pediatr Res. 1999;46(4):461-4.

4. Cooke RJ, Griffin IJ, McCormick K, Wells JC, Smith JS, Robinson SJ, et al. Feeding preterm infants after hospital discharge: effect of dietary manipulation on nutrient intake and growth. Pediatr Res. 1998;43(3):355-60.

5. Cooke RJ, Griffin I, Wells J, Smith J, Leighton M, Robinson S, et al. Formula feeding preterm infants after hospital discharge: 2. Effects on body composition. Pediatr Res. 1996;39(4):306A.

6. Cooke RJ, Griffin I, Wells J, Smith J, Robinson S, Leighton M. Feeding preterm infants after hospital discharge: 1. Effect of type of formula on nutrient intake and growth. Pediatr Res. 1996;39(4):307A.

**Cooper 1988**

1. Cooper PA, Rothberg AD, Davies VA, Argent AC. Comparative growth and biochemical response of very low birthweight infants fed own mother's milk, a premature infant formula, or one of two standard formulas. J Pediatr Gastroenterol Nutr. 1985;4(5):786-94.

2. Cooper PA, Rothberg AD, Davies VA. Three year growth and developmental follow up of very low birthweight infants fed own mother's milk (OMM), a premature infant formula (PF) or one of two standard formulas. Pediatr Res. 1988;23:445A.

**daCunha 2016**

1. da Cunha RD, Lamy Filho F, Rafael EV, Lamy ZC, de Queiroz AL. Breast milk supplementation and preterm infant development after hospital discharge: a randomized clinical trial. J Pediatr (Rio J). 2016;92(2):136-42.

**Dogra 2017**

1. Dogra S, Thakur A, Garg P, Kler N. Effect of Differential Enteral Protein on Growth and Neurodevelopment in Infants <1500 g: A Randomized Controlled Trial. J Pediatr Gastroenterol Nutr. 2017;64(5):e126-e32.

**Fewtrell 2001**

1. Fewtrell MS, Morley R, Abbott RA, Singhal A, Stephenson T, MacFadyen UM, et al. Catch-up growth in small-for-gestational-age term infants: a randomized trial. Am J Clin Nutr. 2001;74(4):516-23.

2. Morley R, Fewtrell MS, Abbott RA, Stephenson T, MacFadyen U, Lucas A. Neurodevelopment in children born small for gestational age: a randomized trial of nutrient-enriched versus standard formula and comparison with a reference breastfed group. Pediatrics. 2004;113(3 Pt 1):515-21.

3. Singhal A, Cole TJ, Fewtrell M, Kennedy K, Stephenson T, Elias-Jones A, et al. Promotion of faster weight gain in infants born small for gestational age: is there an adverse effect on later blood pressure? Circulation. 2007;115(2):213-20.

4. Singhal A, Kennedy K, Lanigan J, Fewtrell M, Cole TJ, Stephenson T, et al. Nutrition in infancy and long-term risk of obesity: evidence from 2 randomized controlled trials. Am J Clin Nutr. 2010;92(5):1133-44.

**Friel 1993**

1. Friel JK, Andrews WL, Matthew JD, McKim E, French E, Long DR. Improved growth of very low birthweight infants. Nutr Res. 1993;13(6):611-20.

**Goldman 1969**

1. Goldman HI, Freudenthal R, Holland B, Karelitz S. Clinical effects of two different levels of protein intake on low-birth-weight infants. J Pediatr. 1969;74(6):881-9.

2. Goldman HI, Liebman OB, Freudenthal R, Reuben R. Effects of early dietary protein intake on low birth weight infants: evaluation at three years of age. J Pediatr. 1971;78:126-9.

3. Goldman HI, Goldman J, Kaufman I, Liebman OB. Late effects of early dietary protein intake on low-birth-weight infants. J Pediatr. 1974;85(6):764-9.

**Jeon 2011**

1. Jeon GW, Jung YJ, Koh SY, Lee YK, Kim KA, Shin SM, et al. Preterm infants fed nutrient-enriched formula until 6 months show improved growth and development. Pediatr Int. 2011;53(5):683-8.

**Lucas 1989**

1. Lucas A, Gore SM, Cole TJ, Bamford MF, Dossetor JF, Barr I, et al. Multicentre trial on feeding low birthweight infants: effects of diet on early growth. Arch Dis Child. 1984;59(8):722-30.

2. Lucas A, Morley R, Cole TJ, Gore SM, Davis JA, Bamford MF, et al. Early diet in preterm babies and developmental status in infancy. Arch Dis Child. 1989;64(11):1570-8.

3. Lucas A, Morley R. Does early nutrition in infants born before term programme later blood pressure? BMJ. 1994;309(6950):304-8.

4. Lucas A, Morley R, Cole TJ, Gore SM. A randomised multicentre study of human milk versus formula and later development in preterm infants. *Arch Dis Child Fetal Neonatal Ed.* 1994;70(2):F141-146.

5. Bishop NJ, Dahlenburg SL, Fewtrell MS, Morley R, Lucas A. Early diet of preterm infants and bone mineralization at age five years. *Acta Paediatr.* 1996;85(2):230-236.

6. Fewtrell MS, Prentice A, Jones SC, et al. Bone mineralization and turnover in preterm infants at 8-12 years of age: the effect of early diet. *J Bone Miner Res.* 1999;14(5):810-820.

7. Morley R, Lucas A. Randomized diet in the neonatal period and growth performance until 7.5-8 y of age in preterm children. *Am J Clin Nutr.* 2000;71(3):822-828.

8. Singhal A, Cole TJ, Lucas A. Early nutrition in preterm infants and later blood pressure: two cohorts after randomised trials. *Lancet.* 2001;357(9254):413-419.

9. Singhal A, Farooqi IS, O'Rahilly S, Cole TJ, Fewtrell M, Lucas A. Early nutrition and leptin concentrations in later life. *Am J Clin Nutr.* 2002;75(6):993-999.

10. Singhal A, Fewtrell M, Cole TJ, Lucas A. Low nutrient intake and early growth for later insulin resistance in adolescents born preterm. *Lancet.* 2003;361(9363):1089-1097.

11. Singhal A, Cole TJ, Fewtrell M, Lucas A. Breastmilk feeding and lipoprotein profile in adolescents born preterm: follow-up of a prospective randomised study. *Lancet.* 2004;363(9421):1571-1578.

12. Isaacs EB, Gadian DG, Sabatini S, et al. The effect of early human diet on caudate volumes and IQ. *Pediatr Res.* 2008;63(3):308-314.

13. Fewtrell MS, Williams JE, Singhal A, Murgatroyd PR, Fuller N, Lucas A. Early diet and peak bone mass: 20 year follow-up of a randomized trial of early diet in infants born preterm. *Bone.* 2009;45(1):142-149.

14. Lewandowski AJ, Lamata P, Francis JM, et al. Breast Milk Consumption in Preterm Neonates and Cardiac Shape in Adulthood. *Pediatrics.* 2016;138(1):07.

**Lucas 1990**

1. Lucas A, Morley R, Cole TJ, et al. Early diet in preterm babies and developmental status at 18 months. *Lancet.* 1990;335(8704):1477-1481.

2. Lucas A, Morley R, Cole TJ. Randomised trial of early diet in preterm babies and later intelligence quotient. *BMJ.* 1998;317(7171):1481-1487.

3. Lucas A, Morley R. Does early nutrition in infants born before term programme later blood pressure? *BMJ.* 1994;309(6950):304-308.

4. Fewtrell MS, Prentice A, Jones SC, et al. Bone mineralization and turnover in preterm infants at 8-12 years of age: the effect of early diet. *J Bone Miner Res.* 1999;14(5):810-820.

5. Morley R, Lucas A. Randomized diet in the neonatal period and growth performance until 7.5-8 y of age in preterm children. *Am J Clin Nutr.* 2000;71(3):822-828.

6. Singhal A, Cole TJ, Lucas A. Early nutrition in preterm infants and later blood pressure: two cohorts after randomised trials. *Lancet.* 2001;357(9254):413-419.

7. Singhal A, Farooqi IS, O'Rahilly S, Cole TJ, Fewtrell M, Lucas A. Early nutrition and leptin concentrations in later life. *Am J Clin Nutr.* 2002;75(6):993-999.

8. Singhal A, Fewtrell M, Cole TJ, Lucas A. Low nutrient intake and early growth for later insulin resistance in adolescents born preterm. *Lancet.* 2003;361(9363):1089-1097.

9. Singhal A, Cole TJ, Fewtrell M, Lucas A. Breastmilk feeding and lipoprotein profile in adolescents born preterm: follow-up of a prospective randomised study. *Lancet.* 2004;363(9421):1571-1578.

10. Isaacs EB, Gadian DG, Sabatini S, et al. The effect of early human diet on caudate volumes and IQ. *Pediatr Res.* 2008;63(3):308-314.

11. Fewtrell MS, Williams JE, Singhal A, Murgatroyd PR, Fuller N, Lucas A. Early diet and peak bone mass: 20 year follow-up of a randomized trial of early diet in infants born preterm. *Bone.* 2009;45(1):142-149.

12. Lewandowski AJ, Lamata P, Francis JM, et al. Breast Milk Consumption in Preterm Neonates and Cardiac Shape in Adulthood. *Pediatrics.* 2016;138(1):07.

13. Isaacs EB, Morley R, Lucas A. Early diet and general cognitive outcome at adolescence in children born at or below 30 weeks gestation. *J Pediatr.* 2009;155(2):229-234.

**Lucas 1996**

1. Lucas A, Fewtrell MS, Morley R, et al. Randomized outcome trial of human milk fortification and developmental outcome in preterm infants. *Am J Clin Nutr.* 1996;64(2):142-151.

**Lucas 2001**

1. Lucas A, Fewtrell MS, Morley R, et al. Randomized trial of nutrient-enriched formula versus standard formula for postdischarge preterm infants. *Pediatrics.* 2001;108(3):703-711.

**Morgan 2011**

1. Morgan C, Herwitker S, Badhawi I, et al. SCAMP: standardised, concentrated, additional macronutrients, parenteral nutrition in very preterm infants: a phase IV randomised, controlled exploratory study of macronutrient intake, growth and other aspects of neonatal care. *BMC Pediatr.* 2011;11:53.

2. Morgan C, McGowan P, Herwitker S, Hart AE, Turner MA. Postnatal head growth in preterm infants: a randomized controlled parenteral nutrition study. *Pediatrics.* 2014;133(1):e120-128.

3. Morgan C, Burgess L. High Protein Intake Does Not Prevent Low Plasma Levels of Conditionally Essential Amino Acids in Very Preterm Infants Receiving Parenteral Nutrition. *JPEN J Parenter Enteral Nutr.* 2015.

4. Morgan C, Burgess L. High protein intake does not prevent low plasma levels of conditionally essential amino acids in very preterm infants receiving parenteral nutrition. *JPEN J Parenter Enteral Nutr.* 2017;41(3):455-462.

5. Tan M, Parry S, Morgan C. Neurodevelopmental outcome in very preterm infants randomised to receive two different parenteral nutrition regimens: the SCAMP nutrition study. *Arch Dis Child.* 2016;101 (Supplement 1):A5.

6. Morgan C PSTM. Neurodevelopmental outcome in very preterm infants randomised to receive two different parenteral nutrition regimens: the scamp nutrition study. *Eur J Pediatr.* 2016;175(11):1516-1517.

7. Morgan C, Parry S, Tan M. Neurodevelopmental outcome at 2.5 years in very preterm infants randomised to receive two different parenteral nutrition regimens at birth: the SCAMP nutrition study. *J Pediatr Gastroenterol Nutr.* 2017;64 (Supplement 1):764.

8. Morgan C, McGowan P, Herwitker S, Hart AE, Turner MA. Early postnatal head growth in very preterm infants: The randomised controlled scamp nutrition study. *J Neonatal Perinatal Med.* 2013;6(2):197.

9. Morgan C, McGowan P, Herwitker S, Hart AE, Turner MA. Preventing early postnatal head growth failure in very preterm infants: The randomised controlled scamp nutrition study. *Arch Dis Child Educ Pract E.* 2013;98:A77-A78.

**O’Connor 2008**

1. O'Connor DL, Khan S, Weishuhn K, et al. Growth and nutrient intakes of human milk-fed preterm infants provided with extra energy and nutrients after hospital discharge. *Pediatrics.* 2008;121(4):766-776.

2. Aimone A, Rovet J, Ward W, et al. Growth and body composition of human milk-fed premature infants provided with extra energy and nutrients early after hospital discharge: 1-year follow-up. *J Pediatr Gastroenterol Nutr.* 2009;49(4):456-466.

3. O'Connor DL, Weishuhn K, Rovet J, et al. Visual development of human milk-fed preterm infants provided with extra energy and nutrients after hospital discharge. *JPEN J Parenter Enteral Nutr.* 2012;36(3):349-353.

4. Karen Weishuhn C. Visual development of premature infants fed human milk containing extra energy and nutrients after hospital discharge. *EPAS 2007.* 2007;<http://www.abstracts2view.com/pas/(5740.4>).

5. O'Connor. Growth and nutrient intakes of human milk-fed premature infants provided with extra energy and nutrients after hospital discharge. *EPAS 2007.* 2007;<http://www.abstracts2view.com/pas/(7720.6>).

**Roggero 2012**

1. Taroni E, Liotto N, Orsi A, et al. [Quality of post-discharge growth in small for gestational age preterm infants: an explorative study]. *Pediatr Med Chir.* 2009;31(3):121-125.

2. Roggero P, Giannì ML, Amato O, et al. Growth and fat-free mass gain in preterm infants after discharge: a randomized controlled trial. *Pediatrics.* 2012;130(5):e1215-1221.

3. Gianni ML, Roggero P, Amato O, et al. Randomized outcome trial of nutrient-enriched formula and neurodevelopment outcome in preterm infants. *BMC Pediatr.* 2014;14:74.

**Svenningsen 1982**

1. Svenningsen NW, Lindroth M, Lindquist B. Growth in relation to protein intake of low birth weight infants. *Early Hum Dev.* 1982;6(1):47-58.

2. Svenningsen NW, Lindroth M, Lindquist B. A comparative study of varying protein intake in low birthweight infant feeding. *Acta Paediatr Scand Suppl.* 1982;296:28-31.

**Tan 2008**

1. Tan MJ, Cooke RW. Improving head growth in very preterm infants--a randomised controlled trial I: neonatal outcomes. *Arch Dis Child Fetal Neonatal Ed.* 2008;93(5):F337-341.

2. Tan M, Abernethy L, Cooke R. Improving head growth in preterm infants--a randomised controlled trial II: MRI and developmental outcomes in the first year. *Arch Dis Child Fetal Neonatal Ed.* 2008;93(5):F342-346.

**Zachariassen 2011**

1. Zachariassen G, Faerk J, Grytter C, et al. Nutrient enrichment of mother's milk and growth of very preterm infants after hospital discharge. *Pediatrics.* 2011;127(4):e995-e1003.

2. Zachariassen G, Faerk J, Esberg BH, et al. Allergic diseases among very preterm infants according to nutrition after hospital discharge. *Pediatr Allergy Immunol.* 2011;22(5):515-520.

3. Zachariassen G. Nutrition, growth, and allergic diseases among very preterm infants after hospital discharge. *Dan Med J.* 2013;60(2):B4588.

4. Zachariassen G, Faerk J, Grytter C, et al. Growth among very premature infants fed human milk fortifier while breastfeeding after hospital discharge. *Acta Paediatr Suppl.* 2009.

5. Toftlund LH, Halken S, Agertoft L, Zachariassen G. Early nutrition and signs of metabolic syndrome at 6 y of age in children born very preterm. *Am J Clin Nutr.* 2018;107(5):717-724.

6. Toftlund LH, Halken S, Agertoft L, Zachariassen G. Catch-up growth, rapid weight growth, and continuous growth from birth to 6 years of age in very-preterm-born children. *Neonatology.* 2018;114(4):285-293.

7. Toftlund LH, Agertoft L, Halken S, Zachariassen G. Improved lung function at age 6 in children born very preterm and fed extra protein post-discharge. *Pediatr Allergy Immunol.* 2019;30(1):47-54
