# Supplementary figures and images for: Impact of macronutrient supplements for children born preterm or small for gestational age on developmental and metabolic outcomes: A systematic review and meta-analysis
Source: PLoS Med. 2019 Oct 30;16(10):e1002952. doi: 10.1371/journal.pmed.1002952 (PMC6821063; doi:10.1371/journal.pmed.1002952)

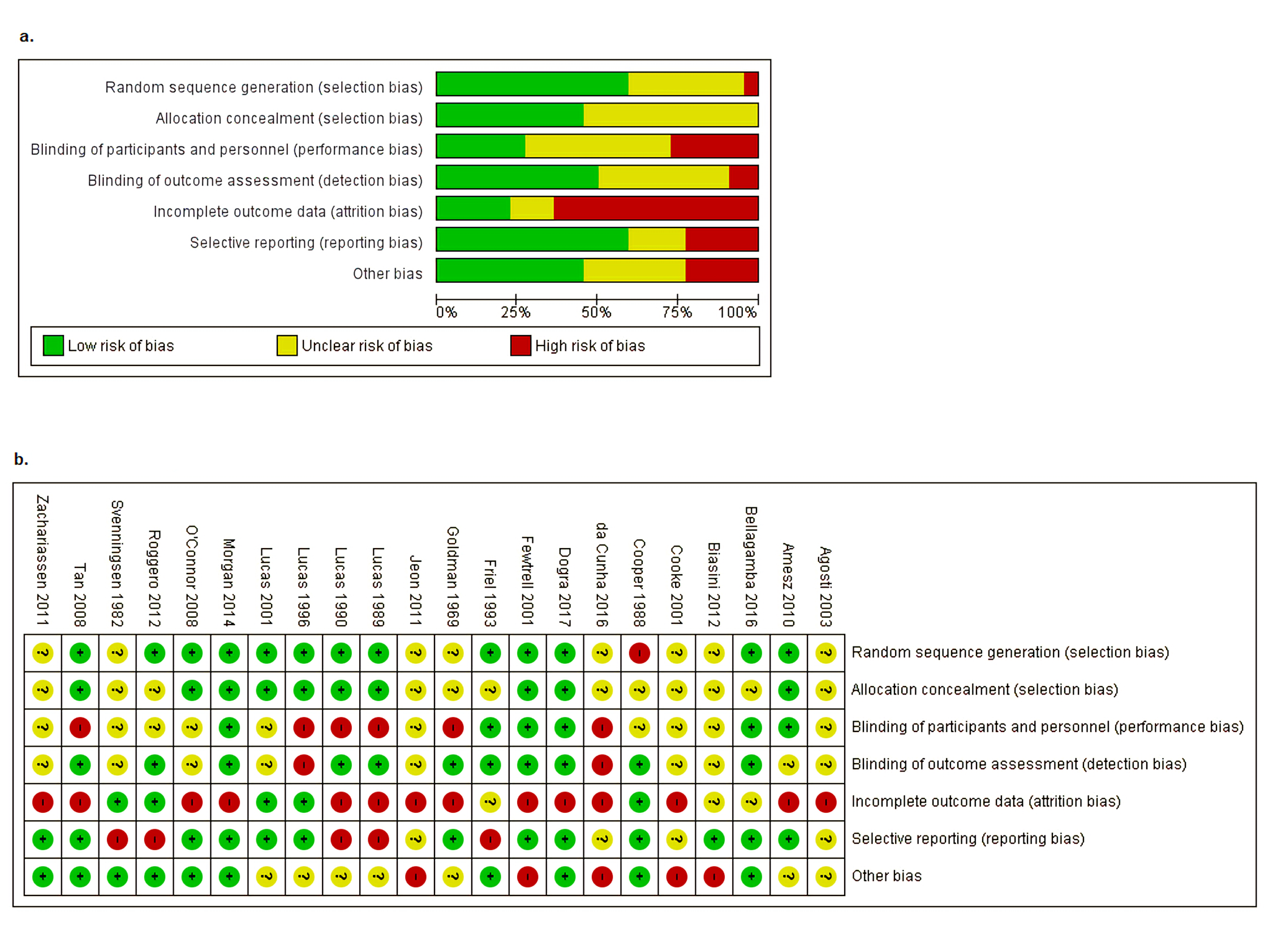

Supplement: S1 Fig — (a) Risk of bias graph: review authors’ judgements about each risk of bias item presented as percentages across all included studies. (b) Risk bias summary: review authors’ judgements about each risk of bias item for each included study. (TIF) [file pmed.1002952.s006.tif]

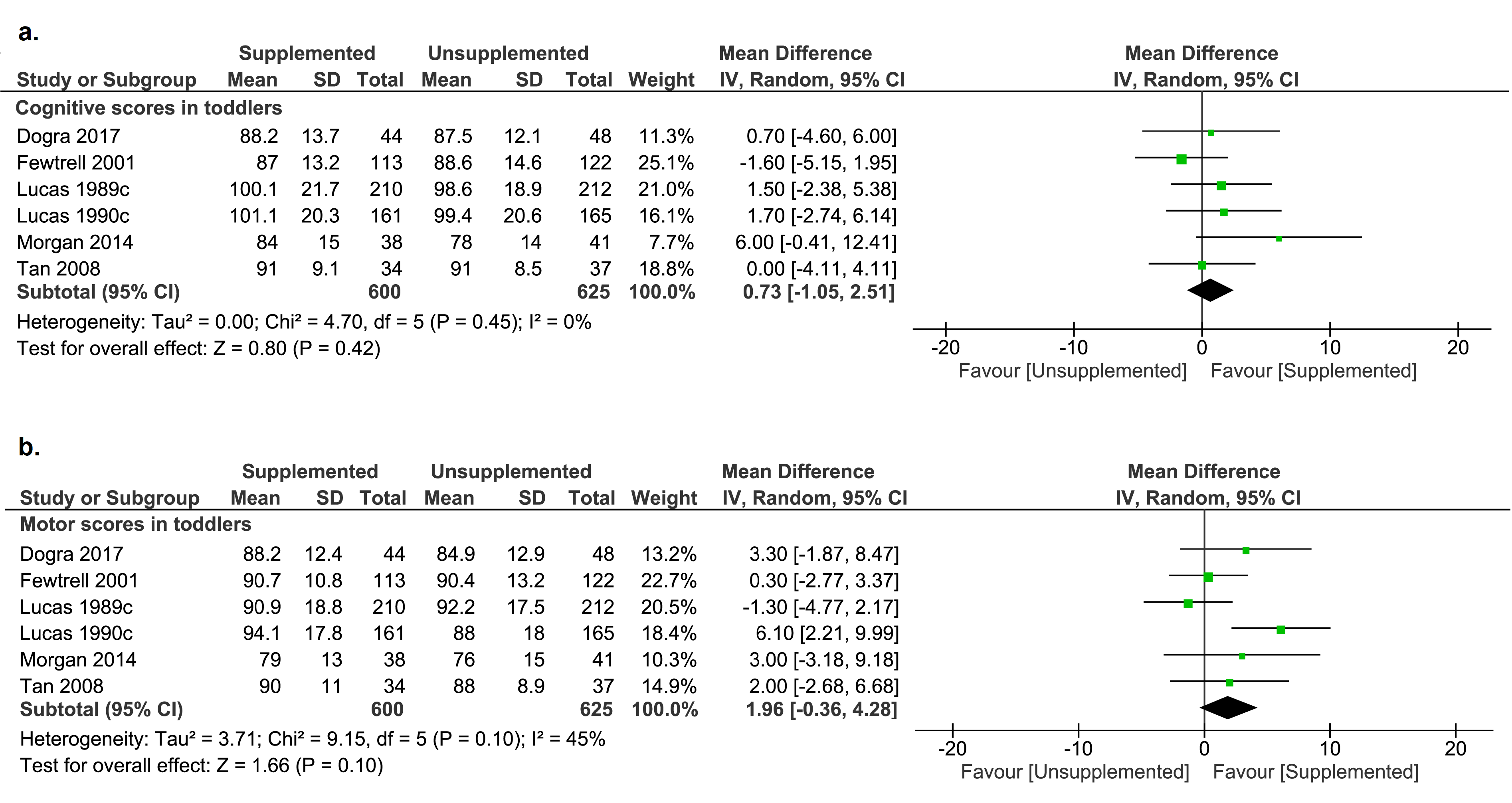

Supplement: S2 Fig — Forest plots of effect of macronutrient supplementation on cognitive scores and motor scores including trials with low risk of bias. (a) Cognitive scores, (b) motor scores. (TIF) [file pmed.1002952.s007.tif]

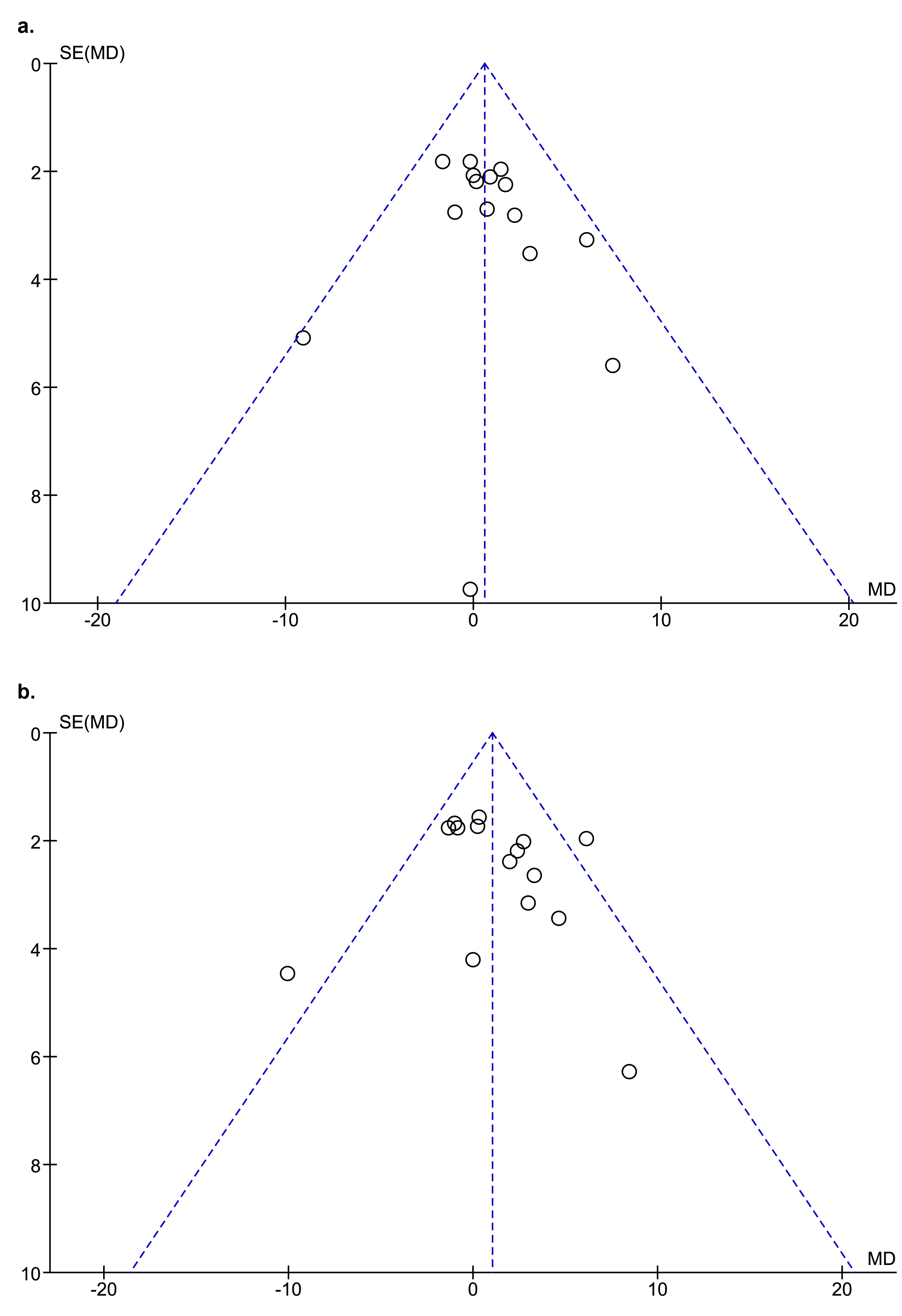

Supplement: S3 Fig — Funnel plots of supplemented versus unsupplemented nutrition for the outcomes of cognitive and motor scores in toddlers. (a) Cognitive scores, (b) motor scores. The middle dashed line indicates the overall MD. The dashed lines either side represent the pseudo 95% CIs. MD, mean difference. (TIF) [file pmed.1002952.s008.tif]

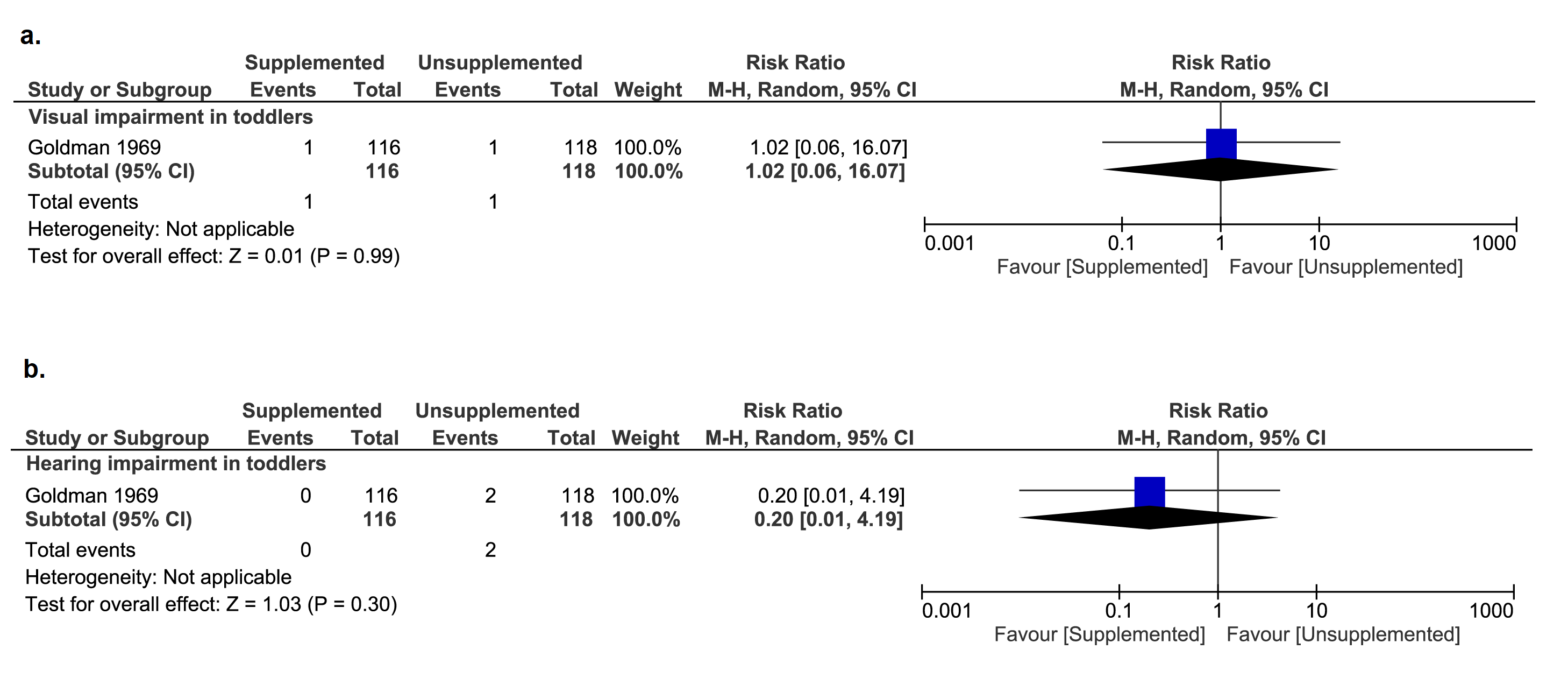

Supplement: S4 Fig — (a) Visual impairment, (b) hearing impairment. (TIF) [file pmed.1002952.s009.tif]

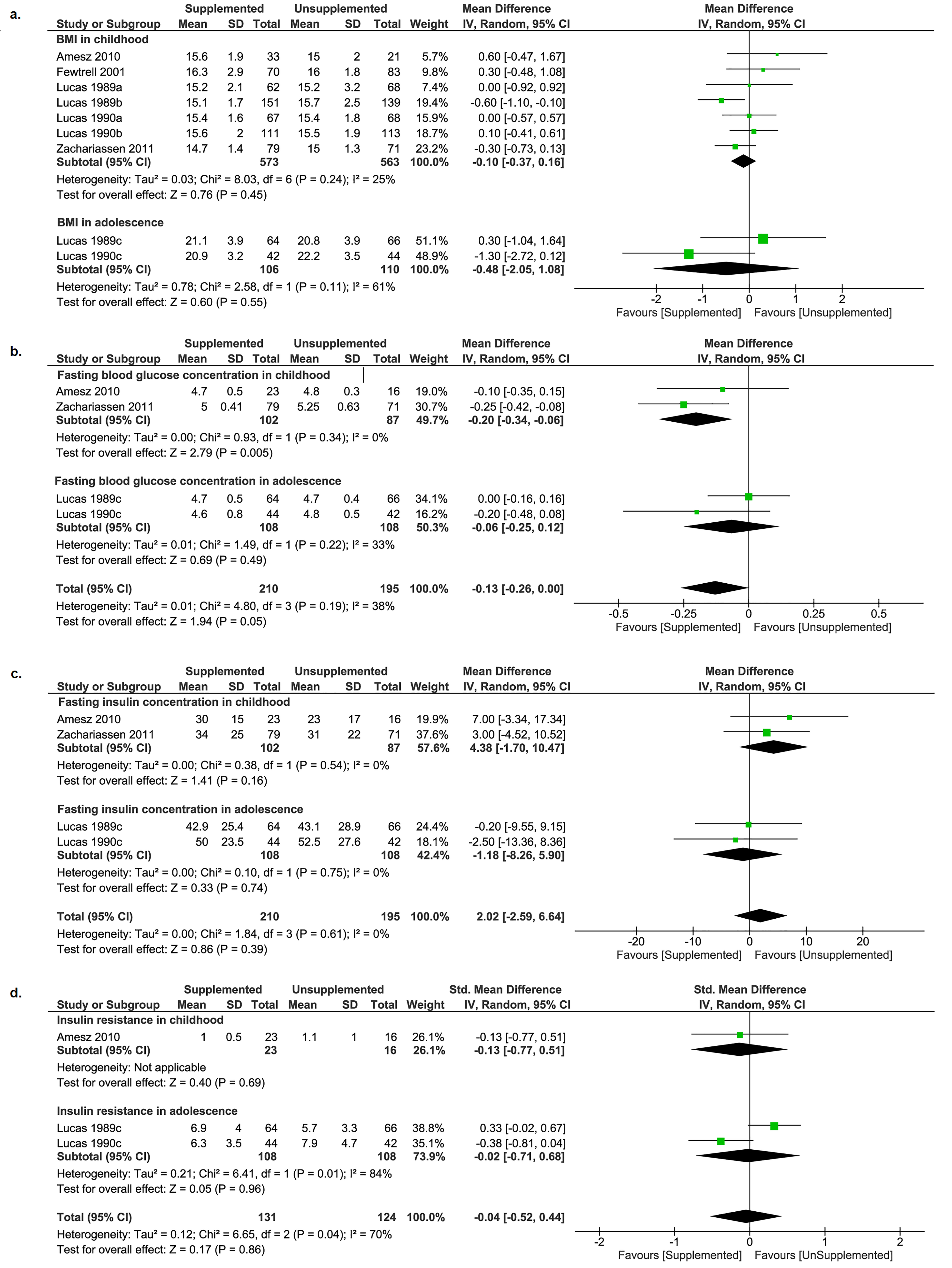

Supplement: S5 Fig — (a) BMI, (b) fasting blood glucose concentrations, (c) fasting insulin concentrations, (d) insulin resistance. BMI, body mass index. (TIF) [file pmed.1002952.s010.tif]

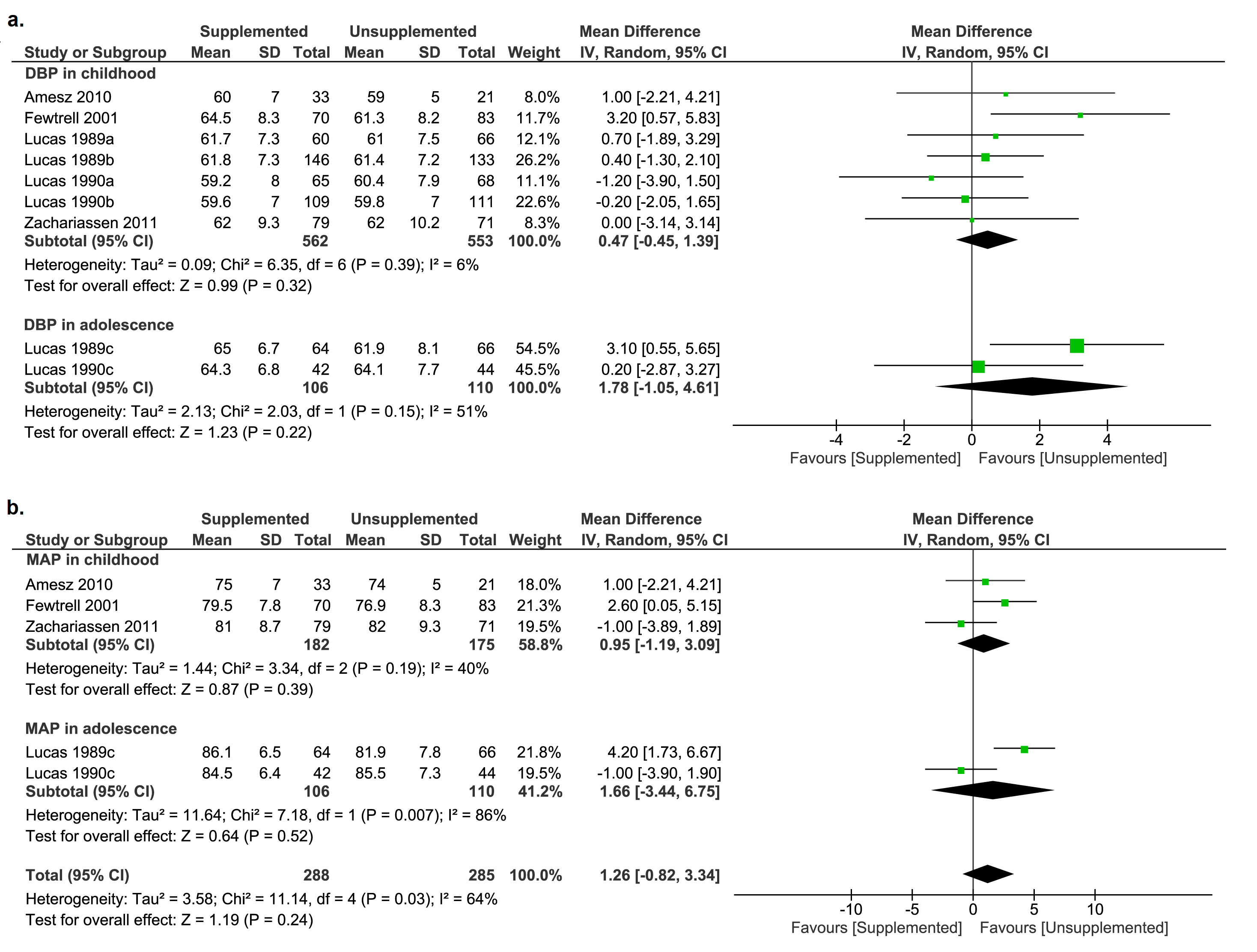

Supplement: S6 Fig — (a) DBP, (b) MAP. DBP, diastolic blood pressure; MAP, mean arterial pressure. (TIF) [file pmed.1002952.s011.tif]

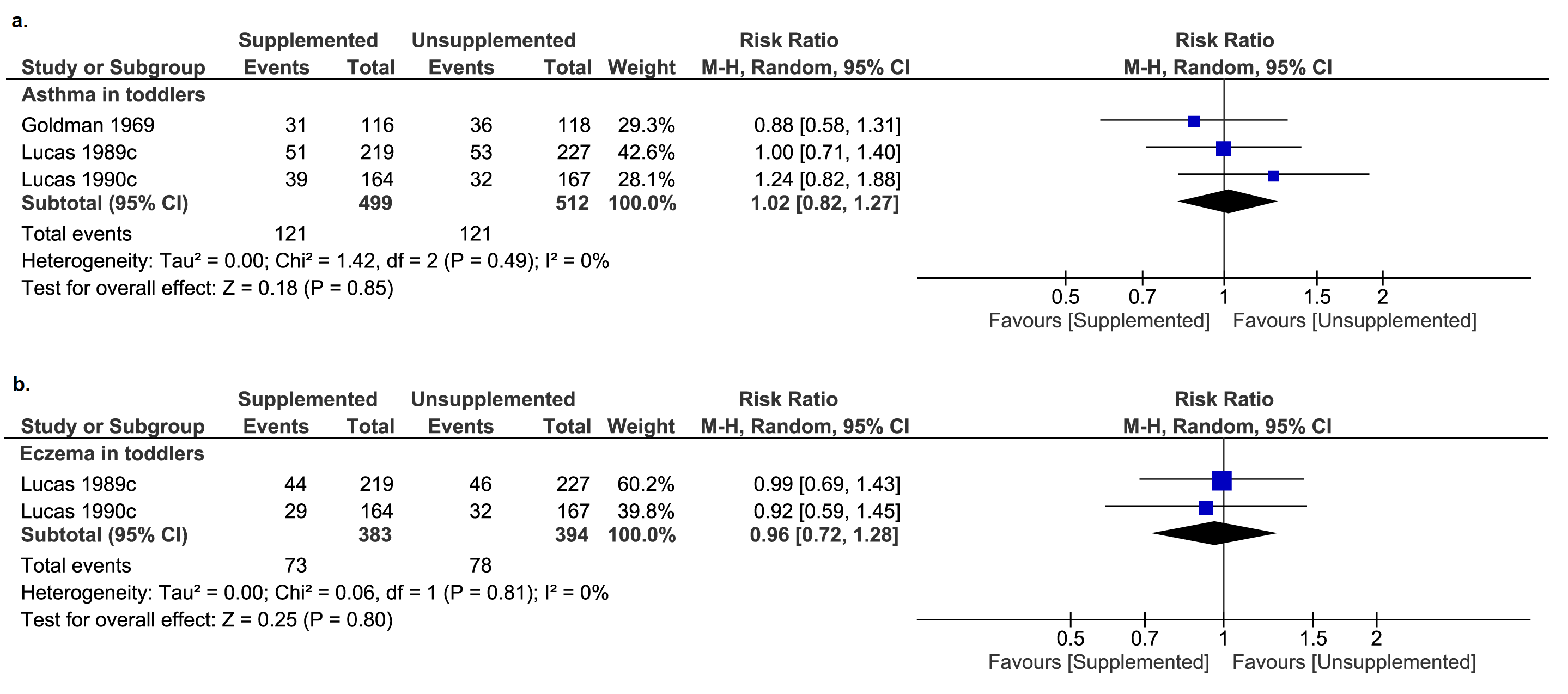

Supplement: S7 Fig — (a) Asthma, (b) eczema. (TIF) [file pmed.1002952.s012.tif]
